# Sensitizing TADF Absorption Using Variable Length Oligo(phenylene ethynylene) Antennae

**DOI:** 10.3389/fchem.2020.00126

**Published:** 2020-02-26

**Authors:** Olga Franco, Marius Jakoby, Rebekka V. Schneider, Fabian Hundemer, Daniel Hahn, Bryce S. Richards, Stefan Bräse, Michael A. R. Meier, Uli Lemmer, Ian A. Howard

**Affiliations:** ^1^Department of Electrical Engineering and Information Technology, Light Technology Institute, Karlsruhe Institute of Technology, Karlsruhe, Germany; ^2^Department of Electrical Engineering and Information Technology, Institute of Microstructure Technology, Karlsruhe Institute of Technology, Eggenstein-Leopoldshafen, Germany; ^3^Laboratory of Applied Chemistry, Institute of Organic Chemistry, Karlsruhe Institute of Technology, Karlsruhe, Germany; ^4^Institute of Organic Chemistry, Karlsruhe Institute of Technology, Karlsruhe, Germany; ^5^Institute of Biological and Chemical Systems - Functional Molecular Systems, Karlsruhe Institute of Technology, Eggenstein-Leopoldshafen, Germany

**Keywords:** thermally activated delayed fluorescence (TADF), time-resolved photoluminescence spectroscopy, transient absorption spectroscopy (TAS), sequence-define oligomer, charge-transfer state (CT-state)

## Abstract

Beyond their applications in organic light-emitting diodes (OLEDs), thermally activated delayed fluorescence (TADF) materials can also make good photonic markers. Time-gated measurement of their delayed emission enables “background-free” imaging in, for example, biological systems, because no naturally-occurring compounds exhibit such long-lived emission. Attaching a strongly-absorbing antenna, such as a phenylene ethynylene oligomer, to the TADF core would be of interest to increase their brightness as photonic markers. With this motivation, we study a sequence of TADF-oligomer conjugates with oligomers of varying length and show that, even when the absorption of the oligomer is almost resonant with the charge-transfer absorption of the TADF core, the antenna transfers energy to the TADF core. We study this series of compounds with time resolved emission and transient absorption spectroscopy and find that the delayed fluorescence is essentially turned-off for the longer antennae. Interestingly, we find that the turn-off of the delayed fluorescence is not caused by quenching of the TADF charge-transfer triplet state due to triplet energy transfer of the lower-lying triplet state to the antenna, but must be associated with a decrease in the reverse intersystem crossing rate. These results are of relevance for the further development of TADF “dyes” and also, in the broader context, for understanding the dynamics of TADF molecules in the vicinity of energy donors/acceptors (i.e., in fluorescent OLEDs wherein TADF molecules are used as an assistant dopant).

## 1. Introduction

Since the initial reports on compounds that displayed thermally activated delayed fluorescence (TADF) (Parker and Hatchard, 1961; Maciejewski et al., 1986; Uoyama et al., 2012), their use as photonic probes and markers has also been considered. In a few examples, TADF molecules can be utilized as temperature and oxygen sensors (Méhes et al., 2012, 2014; Kochmann et al., 2013; DeRosa et al., 2015; Steinegger et al., 2017; Tonge et al., 2020); they have been used as special dyes (optionally incorporated into nanoparticles to negate the effect of environmental quenchers such as oxygen) that enable time-gated background free imaging due to delayed fluorescence (Xiong et al., 2014; Li et al., 2017, 2019; Zhu et al., 2018; Ni et al., 2019; Zhang et al., 2019b) and they have enable photodynamic therapy (Zhang et al., 2016, 2019a). In order to increase the brightness of the delayed emission for sensor or marking applications (or rate of singlet oxygen generation for photodynamic therapy), a strong absorption of incoming photons is desired. The charge-transfer (CT) nature of the absorption of the TADF core means that the photon absorption of the TADF core is rather weak.

**Figure 1:**
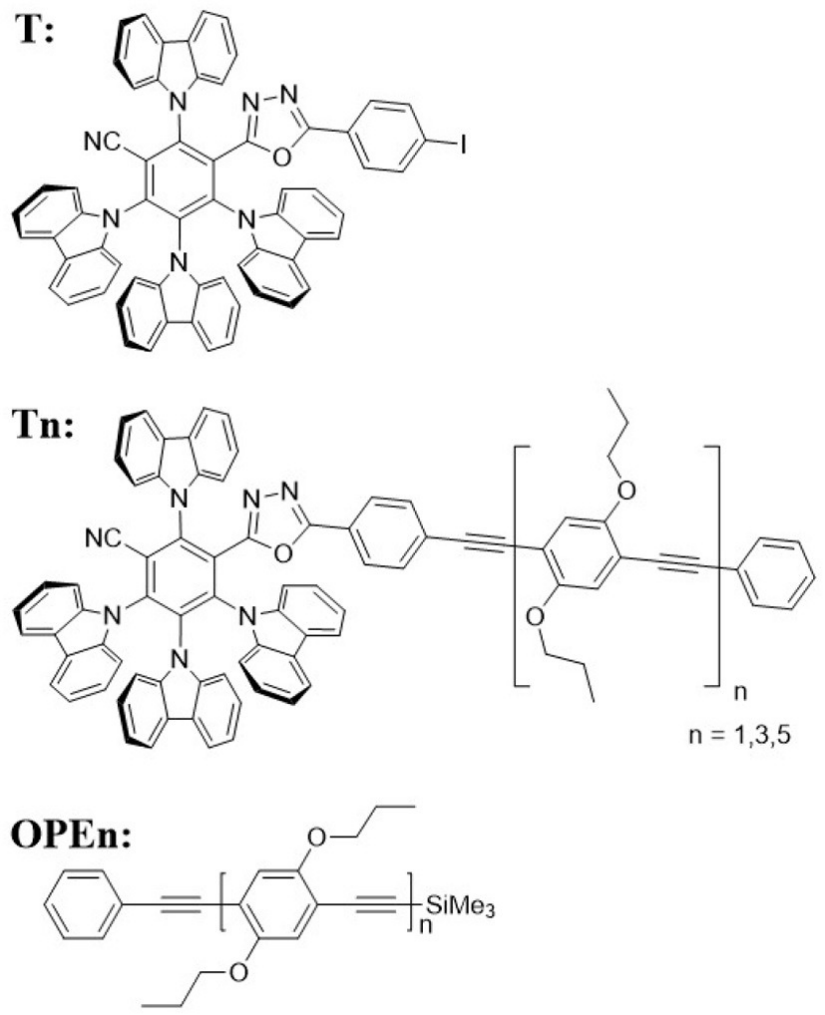
Molecular structure of the investigated compounds.

In this contribution, we consider TADF cores attached to a sequence of strongly-absorbing antennae based on phenylene ethynylene oligomers (OPEs) of varying length (see Figure 1 for the chemical structures of the investigated molecules). By tuning the antenna length, we can achieve strong absorption in resonance with the energy of the TADF CT band. The photons absorbed by the antenna are transferred into the TADF core with good efficiency. However, the delayed fluorescence is also turned off for the compounds with the longer antennae. Examining the kinetics of these systems with time-resolved photoluminescence (PL) and transient absorption, we can exclude quenching of the TADF triplet CT state due to Dexter energy transfer to the lower-lying triplet state on the antenna. Rather, it appears that the reverse intersystem crossing (RISC) rate is diminished in the presence of the antenna. These findings are encouraging for the development of TADF based dyes (and fluorescent OLEDs using TADF assistant dopants) showing that Dexter transfer to a nearby and energetically accessible triplet state is not necessarily kinetically favored and thus might be avoided by chemical design.

## 2. Results and Discussion

### 2.1. Materials

**Figure 2:**
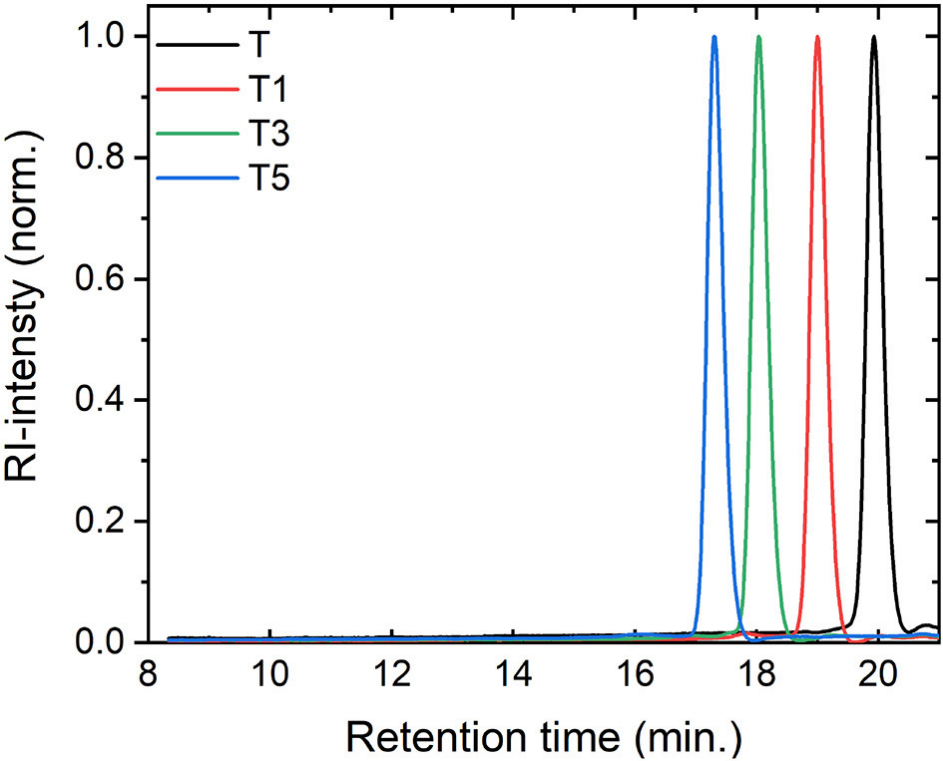
Size exclusion chromatography traces confirming the purity of the compounds.

We attached rod-like monodisperse oligo(phenylene ethynylene)s (OPEs) to a modified 2,4,5,6-tetra(9H-carbazol-9-yl)isophthalonitrile (4CzIPN) core, a compound known for its TADF function (Uoyama et al., 2012). We synthesized a TADF molecule inspired by 4CzIPN, but in which one cyano acceptor group has been replaced by an oxadiazole-phenylene group onto which the OPE can be conjugated, **T** (for synthesis details please refer to Hundemer et al., 2020). Compared to the cyano group, the oxadiazole is a weaker acceptor (Wong et al., 2018). Monodisperse OPEs of varying length were previously synthesized through the use of sequence-defined routes Schneider et al. (2018). Herein the OPE monomer, trimer, and pentamer were coupled to **T** (forming the T-oligomers **T1, T3**, and **T5**, respectively). The chromatography results confirmed the monodispersity of the compounds (cf. Figure 2) The antennae on **Tn** are formed of a chain of **n+2** phenylene units conjugated by **n+1** ethynylene bonds (see Figure 1 for the chemical structures of all the investigated molecules). A detailed report of the synthesis of these compounds can be found in the Supplementary Material.

### 2.2. UV-Vis Absorption and Photoluminescence Spectroscopy

**Figure 3:**
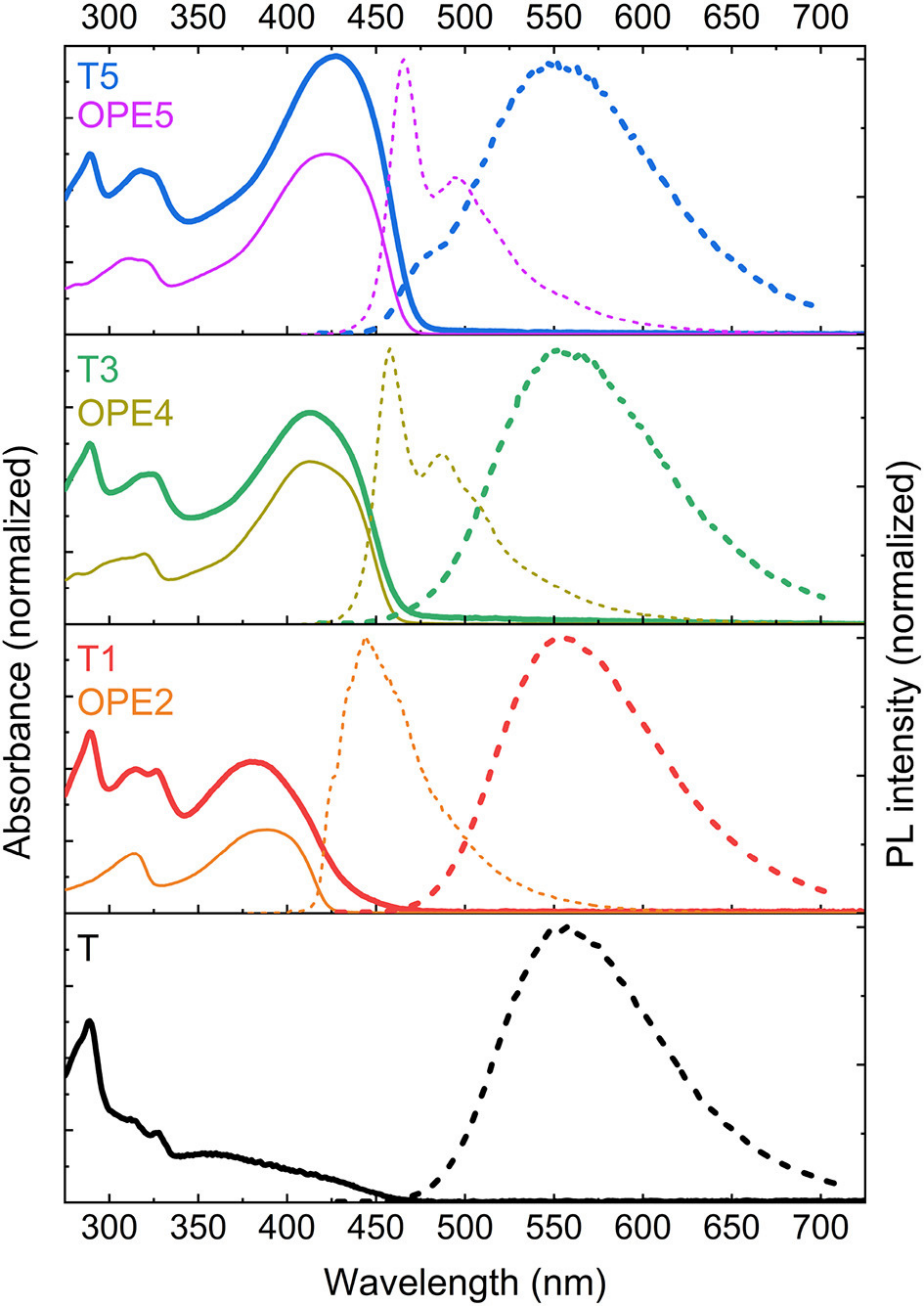
UV-Vis spectra of the investigated compounds (absorption in thick solid lines and emission in thick dashed lines) and their corresponding OPEs (absorption in fine solid lines and emission in fine dashed lines) in chloroform (concentration ≤ 5 · 10^−4^ mol/l). The absorption spectra of **T, T1, T3** and **T5** were normalized to the peak around 290 nm that corresponds to a Cz absorption. The absorption spectra of the OPEs were normalized to the maximum value for **OPE5**. The OPE data were adapted from Schneider et al. (2018), which is an article licensed under a Creative Commons Attribution 4.0 International License (https://creativecommons.org). Specifically, we have calculated the absorbance from the original optical attenuation data. The PL intensity data were used as in the original article.

Figure 3 shows the steady-state absorption and emission spectra of **T, T1, T3**, and **T5**. Alongside, the absorption and emission spectra of the reference OPEs are also shown. The reference OPEs were chosen to have the closest number of conjugated phenyl rings as the antennae on the TADF-conjugates. For **T1** and **T3**, OPEs with the precisely same number of phenylene units are available, whereas for **T5** the OPE has one fewer phenylene ring. In all cases, the agreement between the **Tn** and reference **OPEn** absorption spectra is pronounced, allowing accurate conclusions to be drawn. Upon the addition of an antenna the absorbance spectrum of **T** is modified with an intense low energy band whose peak position and intensity corresponds to that of the reference oligomer (cf. Figure 3). The strong band associated to the antenna greatly influences the absorbance of the TADF-antenna conjugates. Increasing oligomer length increases the oscillator strength of the absorbance and shifts it to lower energies, as expected from π−π^*^ transitions with enhanced conjugation. The increase in the cross-section of the absorption due to the antenna can be easily observed by considering the ratio of the absorbance for the 290 nm peak resulting from the Cz units on the TADF core with the absorbance for the maximum of the bands associated with the antennae. Whereas the CT absorption band for **T** (in the region 340–480 nm) is far weaker than the Cz absorption at 290 nm, the absorption of the longer T-oligomers **T3** and **T5** is stronger at 400 nm (which lies within the CT band) than it is at 290 nm. This is clearly an effect of the antenna. Indeed, strong absorption bands are obtained, with the absorption in the region of the CT band now exceeding the UV carbazole absorption for both **T3** and **T5**. From these data we conclude that the addition of an OPE antenna is an effective way to introduce a strong absorption band, and that for **T5** the oligomer has reached sufficient length that this strong absorption is in resonance with the CT absorption of the original core. Thus, should energy transfer occur from the antenna to the core for **T3** or **T5** they may represent good candidates for TADF-based photonic markers.

In order to investigate whether energy transfer from the antenna to the TADF core is possible, data for the time-integrated PL after excitation at 355 nm were collected, and compared with the PL spectra for the reference OPE compounds. These data are also shown in Figure 3. Although the emission spectrum of the isolated OPE oligomers shifts steadily to the red with increasing length they remain higher in energy than the emission of the unsubstituted molecule **T**. The emission spectra of the TADF-antenna conjugates **T1, T3**, and **T5** remain essentially constant and identical to the spectra of **T** with their peak around 560 nm (a comparison of these spectra on a single plot and their time evolution is also shown in Supplementary Figure 8; from these data molecular aggregation can be ruled out). The observation that the emissions for **T** and **T1, T3**, come from the same state despite stronger absorption from the antenna at the excitation wavelength indicates that there is energy transfer from the antenna to the core. Even for **T5**, the majority of the emitted photons come from the TADF core, although a small high energy shoulder in the emission indicate a minority of singlet states on the antenna can emit before they are transferred in this case. Given this shoulder accounts for only a minor contribution to the overall emission, and also in light of the transient absorption results discussed below, the PL data indicate that for all conjugates energy transfer from the antenna to the TADF core is efficient.

The good transfer to the TADF core from the antennae is achieved because the Stokes shift for **T** is significantly larger than for the individual OPEs. This means that even as the absorption of the antenna becomes similar in energy to the lowest energy CT absorption of the core, the emission of the core is still at a significantly lower energy than that of the antenna. In all cases there is sufficient overlap between the emission of the OPE antenna and the CT absorption of the core to support Förster transfer enabling efficient singlet energy transfer from antenna to core.

For the interested reader, three minor notes regarding the steady-state absorption and emission spectra are made. Firstly, we note that the absorption and emission data for the OPE compounds were previously published Schneider et al. (2018), but reproduced here due to their relevance to the discussion of the new observations. Secondly, we point out that our measurements are made using chloroform as a solvent rather than toluene (commonly used for 4CzIPN) because the TADF was more intense for our molecules in chloroform. This result is consistent with literature findings for 4CzIPN and various carbazole benzonitrile derivatives where the reverse intersystem crossing rate is increased in solvents more polar than toluene (Ishimatsu et al., 2013; Nobuyasu et al., 2016; Hosokai et al., 2018). The decrease in the TADF emission for 4CzIPN in more polar solvents than toluene is attributed to increased non-radiative rates (Ishimatsu et al., 2013). Thirdly, we point out that the Stokes shift for **T** is significantly larger than the Stokes shift observed for 4CzIPN in toluene (Uoyama et al., 2012). There are two effects at play here. On the one hand, the more polar nature of the solvent leads to a greater stabilization of the CT state and a larger Stokes shift. For example the emission peak of 4CzIPN shifts from 507 nm in toluene to 536 nm in dichloromethane (a solvent more polar than chloroform). On the other hand, an increased structural relaxation in the excited state might be introduced by the substitution of the cyano group with the oxadiazole-phenylene group. The emission peak for **T** in toluene is already at 540 nm, significantly red-shifted compared to 4CzIPN. Therefore, the substitution of the cyano group with the weaker accepting oxadiazole group enables a relaxation in the excited state that reduces the energy of the emitted photons and increases the Stokes shift (perhaps supported by the reduced symmetry of the substituted molecule).

### 2.3. Time-Resolved Photoluminescence Spectroscopy

**Figure 4:**
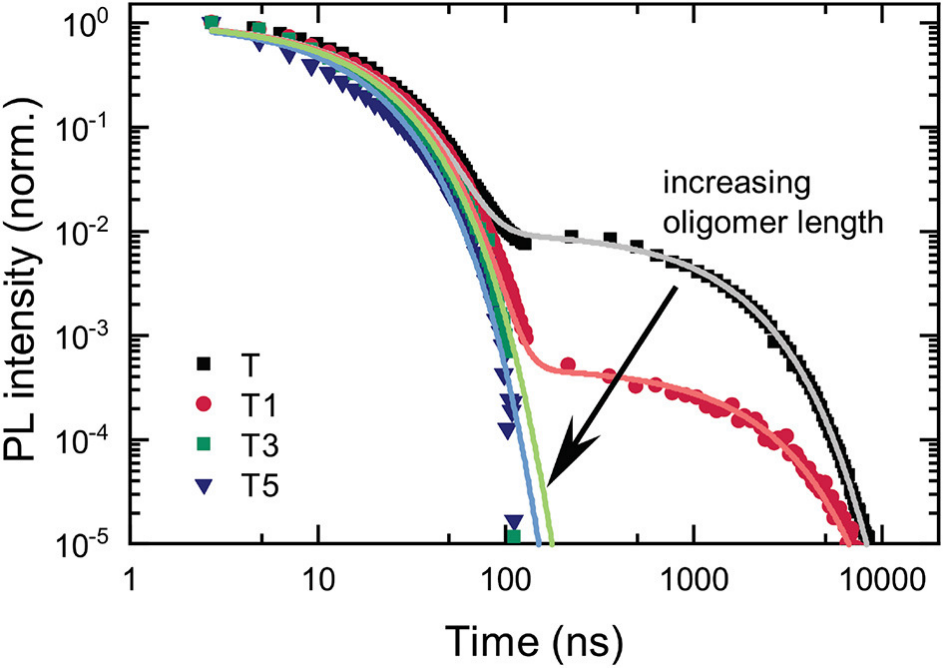
Temporal decay of the PL of the investigated compounds in deoxygenated chloroform (*c* = 5 · 10^−4^ mol/l). Lifetimes and amplitudes of the biexponential fits are stated in Table 1.

We examined the time-resolved emission to establish how the addition of the antenna oligomers affects the prompt and delayed fluorescence emitted from the TADF-antenna conjugates. The temporal evolution of the PL is depicted in Figure 4. Lifetimes obtained from biexponential fits to the data are shown in Table 1. It is immediately apparent that the delayed PL is drastically reduced from **T** to **T1** and suppressed to such a great extent for **T3** and **T5** that no delayed emission can be detected with our sensitive ICCD setup. These results are clearly negative in terms of applying these materials for photonic markers that show strong absorption and delayed emission. Although the absorption is strong on the antenna and energy from the singlet state of the antenna can transfer to the TADF core, once the triplet state is created on the TADF core it is very unlikely to reverse intersystem cross and yield delayed emission. Although this lack of delayed emission precludes the intended application of the molecules, the process by which the delayed fluorescence is turned off is studied in more detail. The results of this investigation are interesting both in terms of understanding how to design molecules for the intended application, and also in terms of their implications regarding the quenching of triplets by fluorescence acceptors in hyperfluorescent systems.

**Table 1:** Prompt and delayed lifetimes (τ_*p*_ and τ_*d*_), and prompt and delayed amplitudes (A_*p*_ and A_*d*_) from biexponential fits of the PL kinetics. Prompt to delayed emission ratio (p/d) calculated using τ_*p*_*A*_*p*_/(τ_*d*_*A*_*d*_).

	**T**	**T1**	**T3**	**T5**
				
τ_p_ /ns	16	16	15	14
τ_d_ /ns	1,200	1,700	NA	NA
A_p_	1	1	1	1
A_d_	1 × 10^−2^	5 × 10^−4^	0	0
p/d	1.5	21	NA	NA
				

The most obvious hypothesis to explain the lack of delayed emission is the introduction of a new loss channel from the TADF triplet CT state upon the introduction of the antenna: namely the transfer of the CT triplet state on the TADF core to a lower-lying triplet state on the OPE antenna. As the singlet-triplet gap on the OPE antenna is around 0.7 eV (Köhler et al., 2002; Köhler and Beljonne, 2004), and the singlet energies of the CT state on the TADF core and the OPE antenna are similar, the triplet energy of the OPE antenna must lay significantly below the energy of the CT triplet state on the TADF core. Therefore, the transfer of the CT triplet to the triplet state on the antenna is energetically possible.

However, none of the data we collected are consistent with this hypothesis. This is examined in greater detail in the section below presenting the transient absorption. Already the data shown in Figure 4 are inconsistent with the hypothesis of transfer of the CT triplet to a triplet state localized on the OPE. The prompt and delayed lifetimes along with the ratio of the prompt to delayed emission obtained from biexponential fits of the data shown in Figure 4 are presented in Table 1. In TADF molecules, the lifetime of the delayed fluorescence reveals the decay of the charge-transfer triplet state population. If a new loss channel was introduced to the charge-transfer triplet population, the initial intensity of the delayed PL would be similar to that without the loss channel (i.e., the intensity of the delayed PL at 100 ns would be similar) but the lifetime of the delayed PL would be substantially shorter. However, these are not the observations made in the data. Rather the opposite, between **T** and **T1** the intensity of the delayed PL in the first instance (at 100 ns) is substantially decreased, and the lifetime of the delayed PL is not shortened (in fact it slightly increases in **T1**). The observations in the data rather suggest that the RISC rate is decreased, decreasing the intensity of the delayed PL from a similar concentration CT triplet states.

In order to visualize this argument, simulated PL transient showing the prompt and delayed emission are obtained for three solutions to the rate equations for the singlet and triplet CT populations on the TADF core. The first solution is the reference case, with typical values from the literature used for the kinetic parameters. In the second case, an extra decay channel for the CT triplet state is added to express a transfer of the CT triplet state to a triplet state on the antenna. In the third case, the rate of RISC is reduced by a factor of 20. The rate equations are shown in Equation (1), where *c*_s_ and *c*_t_ are the concentrations of CT states in the singlet and triplet manifold respectively, *k*_ISC_ is the intersystem crossing rate, *k*_rad_ is the radiative decay rate from the CT singlet state (note non-radiative decay from the singlet state to the ground state is neglected, as common in the literature), *k*_RISC_ is the reverse intersystem crossing rate, and *k*_nr+trans_ is the total rate of states exiting the CT triplet population due to non-radiative decay to the ground state or transfer to the triplet state on the antenna.

(1a) $$\begin{array}{r} \frac{\partial }{\partial t}c_{\text{s}}=-\left(k_{\text{ISC}}+k_{\text{rad}}\right)c_{\text{s}}+k_{\text{RISC}}\cdot c_{\text{t}}, \end{array}$$

(1b) $$\begin{array}{r} \frac{\partial }{\partial t}c_{\text{t}}=-\left(k_{\text{RISC}}+k_{\text{nr+q}}\right)c_{\text{t}}+k_{\text{ISC}}\cdot c_{\text{s}}, \end{array}$$

**Figure 5:**
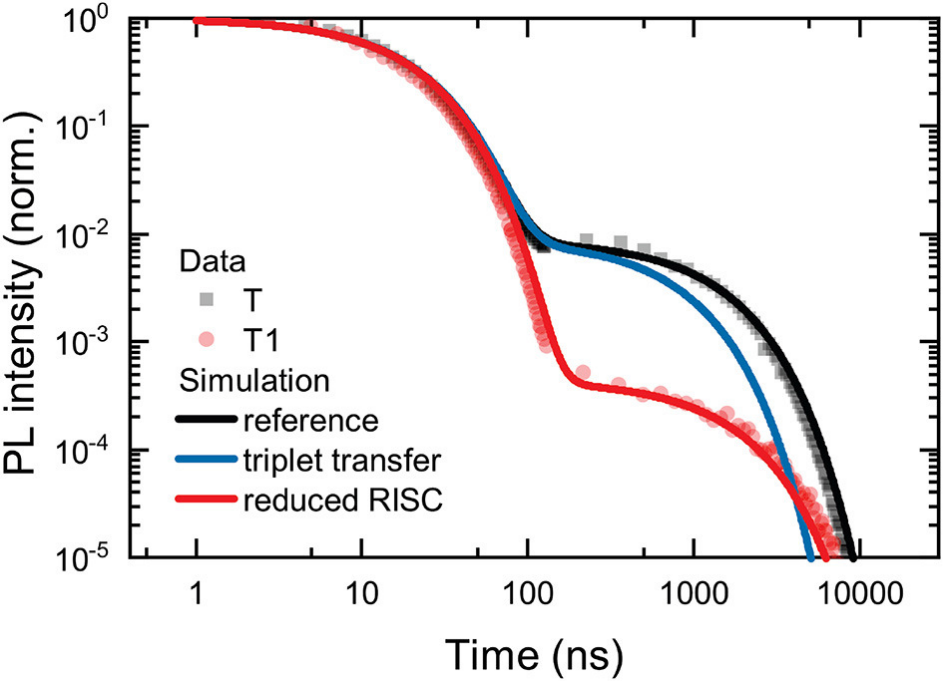
Simulated PL transients for the reference, triplet transfer, and reduced RISC models described in the text. The reference and reduced RISC models agree with the observed data for **T** and **T1**, respectively. The triplet transfer model does not agree with the observed data.

The parameters used for the reference simulation are: loss of CT triplet population due to triplet transfer to antenna and decreased RISC rate. They are shown in Table 2. The parameters are chosen to be consistent with the literature values for 4CzIPN (Uoyama et al., 2012; Yurash et al., 2019), with slight adjustment (slight increases in *k*_rad_, *k*_ISC_, and *k*_nr+q_) so that the reference simulation mimics the kinetics observed for **T**. The results of the simulations are compared with the data for **T** and **T1** in Figure 5. The parameters used for the reference simulation reproduce well the observed PL transient for **T**. However, the addition of a significant extra channel for loss of the CT triplet state, for example caused by transfer of the CT triplet to the oligomer triplet, is not able to explain the observed data for **T1**. In the simulation labeled “triplet transfer,” *k*_nr+q_ is doubled, to represent significant population of the CT triplet transferring to the antenna. As described above, although the total delayed PL is significantly reduced in the triplet transfer model, the initial delayed emission (around 100 ns) is hardly affected. The simulation labeled “reduced RISC,” on the other hand, fits the observed data for **T1** very well. The reduced RISC simulation differs only from the reference case in that *k*_RISC_ is reduced by a factor of 20.

**Table 2:** Parameters used for simulation of PL transients.

	**Reference**	**Triplet transfer**	**Decreased RISC**
*k*_rad_/s^−1^	1.3·10^7^	1.3·10^7^	1.3·10^7^
*k*_ISC_/s^−1^	3.9·10^7^	3.9·10^7^	3.9·10^7^
*k*_RISC_/s^−1^	6·10^5^	6·10^5^	3·10^4^
*k*_nr+q_/s^−1^	6·10^5^	1.2·10^6^	6·10^5^

### 2.4. Transient Absorption Spectroscopy

The results of the transient PL experiments confirm that it is a change in the *k*_*RISC*_ that limits the delayed emission from **T1** through **T5**. In order to substantiate this hypothesis (and gain insights into the intermediate states involved), we investigated the excited-state dynamics of the molecules **T**, **T1**, and **T5** using transient absorption spectroscopy (TAS). We measured the change in transmission (ΔT/T) of the samples in the wavelength range of 520–880 nm induced by a previous excitation pulse with a wavelength of 355 nm. Details of the transient absorption setup are given in the experimental section. We note that the time resolution is sub-nanosecond and the excitation fluence was kept under 20nJcm^−2^. To ascertain that all kinetics presented are caused by linear processes, and that the sample was not degraded during the investigation, measurements at a sequence of excitation fluences were taken.

**Figure 6:**
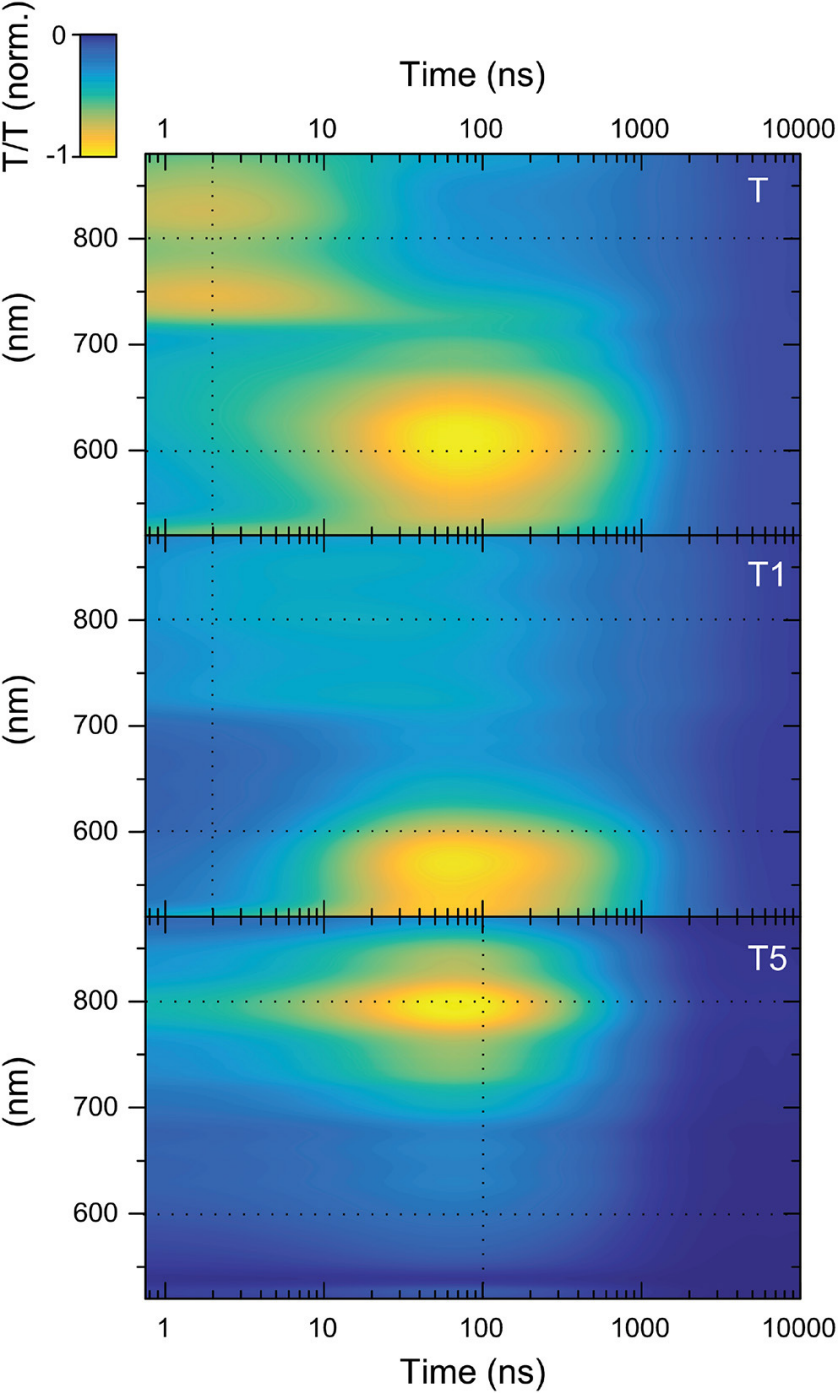
TAS contour maps of the T-oligomers **T, T1**, and **T5**. The black dashed lines correspond to the positions of the kinetics shown in Figure 7.

Figure 6 shows the TAS contour maps of the three samples under study for delay times between the pump and probe beams of 0.8 ns to 10 μs. Immediately, a difference between **T5** and the other two samples is apparent. For **T** and **T1**, two distinct photo-induced absorption (PIA) bands are visible whose maxima occur at significantly different points in time: (i) a band centered around 800 nm present at early times (<5 ns) and (ii) a PIA band centered around 600 nm which rises within the first 100 ns after excitation. For **T** and **T1** the data matrix is rank 2. However, this is not the case for **T5**. There is only one feature visible for the case of **T5** (for **T5** the data matrix is rank 1).

**Figure 7:**
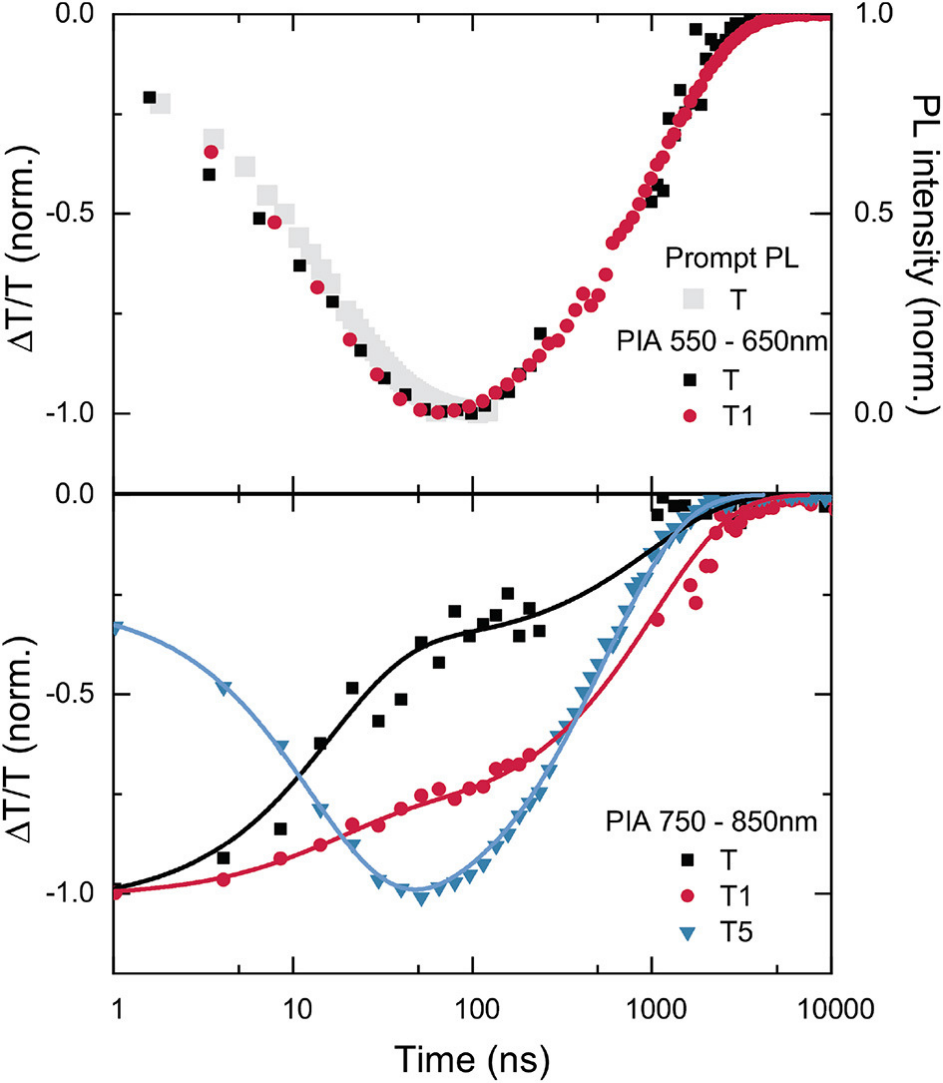
TAS kinetics of the T-oligomers **T, T1**, and **T5** for the integrated wavelength regions 550–650 nm **(top)** and 750–850 nm **(bottom)**.

Already at this level of analysis, these data again provide evidence against the hypothesis that the reduction in delayed emission in **T1**, **T3**, and **T5** stems from triplet transfer from the triplet CT state of the TADF core to a localized triplet on the OPE antenna. If the CT triplet excitations were transferred to the OPE oligomer, this should happen most favorably in **T5**. In **T5** a new spectral feature should emerge after ISC that corresponds to the excited-state absorption of an OPE triplet while the initial excited-state absorption due to the TADF-core CT state should disappear. This is inconsistent with the observed data matrix for **T5**. The observations show that the data matrix for **T5** is rank 1 (meaning that it can be expressed by the outer product of only one spectral vector with a single time-evolution vector). This means that only a single-excited state species is responsible for the entire TA surface for **T5**. This species cannot be an OPE triplet for two reasons. Firstly, the signal is there immediately after excitation. Although the signal grows stronger with time in the first 100 ns, the signal height is roughly 25% of its maximum immediately (cf. lower panel of Figure 7). There is no possibility that such a significant population of triplet states on OPE could be created immediately upon excitation. The second argument against this feature being related to a triplet state on the OPE is its lifetime. The triplet lifetime of poly(p-phenylene ethynylene) was able to be easily determined as it was one of the first materials to show delayed emission due to triplet-triplet annihilation (Partee et al., 1999). This delayed emission allowed a triplet lifetime of 200 μs to be estimated for powder samples (Partee et al., 1999). Later pulse radiolysis measurements agreed well with this, finding a triplet lifetime of 300 μs in deoxygenated toluene solution. We also note that the triplet induced absorption for poly(p-phenylene ethynylene) shows a strong feature at 780 nm. For this reason, we can be confident that we would see signals from the triplet exciton species on the OPE in the spectral range that we observe in case OPE triplet excitons were created.

We now examine the transient absorption data in more detail. A comparison with the detailed TAS study of Hosokai et al. (2017) on 4CzIPN supports the assignment of the band at 600 nm that grows in within the first 100 ns to be associated with a local triplet state on the pthalonitrile core of the molecule (^3^LE). Such bands are only observed for **T** and **T1**. We note that this band is shifted between the two samples. For **T**, the band is centered at 600 nm, but for **T1**, it is blue shifted to 550 nm. This shift in the induced absorption band suggests a shift in the energy levels of the ^3^LE between **T** and **T1**. For **T5**, this band is either entirely absent, or significantly shifted in energy. The development of the intensity of this PIA as a function of time is shown in Figure 7, where it is also compared to the decay of the prompt PL. The growth of the PIA and the decay of the prompt PL exactly coincide, reinforcing the conclusion that the species responsible for the PIA is a direct product of ISC from the CT singlet state.

The energetic resonance of the CT states with ^3^LE is proposed to be crucial for enabling RISC (Etherington et al., 2016; Evans et al., 2018). The ^3^LE can couple to the CT singlet state, allowing the CT triplet to return to the CT singlet with the help of this local triplet intermediate. Therefore, the observation of a local triplet absorption band growing in with 1/(*k*_ISC_ + *k*_rad_) has been observed to be critical for efficient RISC (Hosokai et al., 2017). The presence of this band suggests that the triplet CT efficiently mixes with the local triplet state. The upper panel of Figure 7 shows how this induced absorption grows in for **T** suggesting that mixing dominates the TA spectra after 100 ns. In **T1** it is slightly less dominant with respect to the 800 nm feature (and also shifted). For **T5** this feature is absent and the 800 nm feature dominates the spectrum at later times. Thus, we conclude that there is a correlation between the growth of a strong PIA, likely stemming from a ^3^LE state close to resonance with the CT triplet state, and a reasonable RISC rate. The observations are consistent with our conclusions that it is a reduced RISC rate that is responsible for the reduction of delayed PL. The similar lifetime of this PIA signal from **T** and **T1** again speaks against the hypothesis of quenching of the CT triplet state by transfer to a lower lying triplet on the antenna.

There is a second clear feature in all of the TA surfaces. The PIA around 800 nm can be assigned to an absence of electron density on the Cz units as a consequence of a CT state formation as it closely resembles the induced absorption for Cz cations (Yamamoto et al., 1989; Tsujii et al., 1990). This induced absorption associated with a reduction of electron density on the Cz units is present for all times in all samples; however, the intensity of this induced absorption does change upon ISC in all samples. The lower panel of Figure 7 depicts the evolution of this PIA due to the Cz reduced electron density in the range 750–850 nm. For **T**, the PIA in this region decreases by more than 50% with the same lifetime as the prompt decay. This indicates that for **T**, the transition from the singlet to the triplet CT state is accompanied by a reduction in the PIA caused by a decreased electron density on the carbazole units. This could be explained by a sufficiently small difference in energy between the CT triplet state and a ^3^LE state, that allows the excited state to spend time in the ^3^LE once intersystem crossing has occurred (and thus reducing the amount of time spent in the CT state which causes the 800 nm PIA). Such an explanation is consistent with the work of Etherington et al., who found a changing ratio of the induced absorption of the CT triplet to the ^3^LE state as the energetic gap between these two states was varied (Etherington et al., 2016). Returning to our data, for **T1** the PIA in this region is also reduced on the same time scale as the prompt PL decay. However, in this case the reduction is less than half as large as it was for **T**. This could indicate that again, once the CT state in **T1** has undergone intersystem crossing, it can spend time in a ^3^LE state. However, it spends much less time in the ^3^LE, and consequently more time in the CT triplet state. For this reason, the PIA that stems from a reduction in electron density on the carbazoles (in the CT state) is better preserved in this case. For **T5**, the kinetics are notably different. The PIA in the 800 nm region actually increases with the same rate as the prompt PL lifetime. Although the physical mechanism causing this increase in the strength of the PIA associated with the CT state is not able to be determined with certainty, this observation of a strong CT-associated PIA for **T5** is certainly consistent with lack of population of a ^3^LE in this case (and its negligible rate of RISC).

### 2.5. Low-Temperature Photoluminescence

Finally, we investigated the emission spectra of the compounds at cryogenic temperatures. A comparison of the emission spectrum at room temperature and at 77 K is often used to estimate the singlet-triplet splitting in the CT state of TADF molecules. Also, if the CT triplet could transfer its energy to lower-energy triplet states on the antenna, the phosphorescence from these lower energy triplet states should become observable at low temperature. Comparisons of the room temperature and cryogenic photoluminescence spectra are shown in Supplementary Figures 9–11. Consistent with our observations above, in no case is a low-energy phosphorescence (from a triplet on an antenna) observable. The phosphorescence spectra is not significantly altered between the compounds. In conjugation with their similar fluorescence spectra, this indicates that the energetic levels of the CT state on the TADF core are not drastically altered. From the onsets of the fluorescence and phosphorescence spectra at 77 K, we estimate similar values for the singlet-triplet splittings of T, T1, and T3, [Δ*E*_*ST*_(**T**) = 0.02 eV, Δ*E*_*ST*_(**T1**) = 0.01 eV, and Δ*E*_*ST*_(**T3**) = 0.02 eV]. Despite these similar values for Δ*E*_*ST*_ across the compounds, the previous sections demonstrate that the rate of RISC differs significantly amongst the series.

## 3. Conclusions

We have investigated a sequence of molecules composed of a TADF core conjugated to an OPE antenna whose length varies across the sequence. We find that an OPE antenna of appropriate length can bestow strong photon absorption on the molecule in the spectral region wherein previously only the weak CT absorption was present. This increase of the molar extinction coefficient in the visible by a factor of 5 would assist the application of TADF molecules as photonic markers offering background-free delayed emission. However, although we find that energy transfer from the singlet state of the antenna to the CT singlet of the TADF core can be quantitative, we also find that the delayed emission becomes greatly weakened by the attachment of a short OPE antenna, and completely suppressed for longer antennae.

At first thought, the most probable hypothesis for this turn-off of the delayed emission would be Dexter transfer from the CT triplet state of the TADF core to the lower-lying triplet state present on the OPE antenna. Such a quenching mechanism is also thought to play a role in the TADF-assisted fluorescent OLEDs when the fluorescent dopant is present at too high concentrations. Surprisingly, we find that this simple hypothesis is not consistent with the observed data (neither the time-resolved PL nor the TA data). Rather, the data show that a reduction of the rate of RISC occurs upon the addition of the OPE tails, and suggest that this reduction in RISC rate may be due to a change in the position of a ^3^LE. Certainly, the TA of these blends presents interesting insights into the changing nature of the intermediate species present for a sequence of molecules over which the delayed emission is turned off.

This work suggests that further work to obtain strong delayed emission from TADF core-antenna combinations is not futile but that further work is needed to understand how RISC rates can be maintained upon addition of the antenna. In this regard, a study of more symmetric TADF core dual antenna conjugates may be a fruitful avenue to explore. In general, these results also highlight that careful observation and understanding of Dexter transfer from a TADF molecule to nearby acceptors is an area of continued interest for designing application-optimized TADF materials.

## 4. Experimental

### 4.1. Methods

#### 4.1.1. UV-Vis Absorption

We used a commercial UV-Vis spectrometer (Lambda1050, Perkin Elmer Inc.) to collect the absorption spectra.

#### 4.1.2. Photoluminescence

For the PL measurements (both steady state and time resolved) we used a gated camera setup. As excitation source we used a diode-pumped solid state (DPSS) pulsed laser (Picolo-10, Innolas GmbH) with 0.8 ns pulses at 355 nm and a repetition rate of 5 kHz. The emission was detected with a spectograph (Acton SpectraPro-2300, Princeton Instruments Inc.) coupled to an intensified CCD camera (PiMax4, Princeton Instruments Inc.).

#### 4.1.3. Transient Absorption

TA spectra were recorded by using a pump-probe setup. The setup has two modes of operation: a short-delay mode, for delays between pump and probe pulses in the range from 100 fs to 2 ns, and another one to work in long-delay mode, for delays longer than 2 ns. A commercial Ti:Sa amplifier with 100 fs width pulses at 800 nm and operating at a repetition rate of 1 kHz (Spitfire Pro XP, Spectra Physics, Newport Corp.) is split into two beams. The probe beam is directed to a 2 mm thick sapphire crystal to generate white light pulses (450–1,000 nm) and then to the sample. The pump beam passes first through a light chopper that reduces its repetition rate to the half and then through a barium borate (BBO) crystal that generates second harmonics resulting in a 400 nm pulse with a repetition rate of 500 Hz. The pump beam passes then through a delay stage that consists of a stage with a mirror combination that can be precisely driven over 600 mm to increase or decrease the light path of the pump pulse. The so-delayed pump pulse is then directed to the sample. The light transmitted through the sample is then detected with a spectograph (Acton SpectraPro-300i, Princeton Instruments Inc.) coupled to an intensified CCD camera (PiMax 512, Princeton Instruments Inc.). Adjacent spectra correspond to the transmission of the sample with and without pump pulse and are used to calculate $\frac{\Delta T}{T}$. To work in long-delay mode, the pump pulses are provided by a pulsed DPSS laser (Picolo-AOT MOPA, Innolas GmbH) with 0.8 ns pulses at 355 nm and a repetition rate of 500 Hz. The delay between pump and white light pulses is controlled by an electronic delay generator (DG535, Stanford Research Systems Inc.).

## Data Availability Statement

All data are provided in the experimental section of the article/Supplementary Material. Original data are available upon request from the authors.

## Author Contributions

OF performed the steady-state absorption and PL experiments and the time-resolved PL experiments. MJ performed the simulation of the PL transients and the transient absorption experiments. RS synthesized the T-oligomers (**T1**, **T3**, and **T5**) and prepared the samples. FH synthesized the TADF molecule **T**. DH synthesized the T-oligomers (**T1**, **T3**, and **T5**) and prepared the samples. IH performed the time-resolved PL, the transient absorption experiments, and the simulation of the PL transients. All authors contributed to the discussion and writing of the manuscript.

### Conflict of Interest

The authors declare that the research was conducted in the absence of any commercial or financial relationships that could be construed as a potential conflict of interest.
