# Supplementary material for: Sensitizing TADF Absorption Using Variable Length Oligo(phenylene ethynylene) Antennae
Source: Front Chem. 2020 Feb 26;8:126. doi: 10.3389/fchem.2020.00126 (PMC7054278; doi:10.3389/fchem.2020.00126)
Supplement: Supplementary file 1 [file Data_Sheet_1.pdf]

Supplementary Material

1 Synthesis and chemical characterization of the conjugated oligomer-TADF-conjugates

Our novel TADF-oligomer-conjugates are based on literature-known 1,2,3,5-tetrakis(carbazole-9-yl)-4,6-dicyanobenzene (4CzIPN) and literature-known oligo(phenylene ethynylene)s. The denotation of the oligomer-TADF adducts is dependent on the DP of the connected oligomer, *e.g.* **T1** for the monomer-TADF adduct. Overall, a library of three oligomer-TADF adducts was synthesized: **T1**, **T3** and **T5** (Supplementary Figure 1).

1.1 Materials

The following chemicals were used as received: bis(triphenylphosphine)palladium(II) dichloride (≥99%, Sigma Aldrich), copper(I) iodide (≥99.5%, Sigma-Aldrich), ammonium chloride (≥99%, BASF), anhydrous tetrahydrofuran (≥99.9%, Sigma-Aldrich), sodium sulphate (>99%, Sigma Aldrich), dichloromethane (HPLC-grade, VWR Chemicals), dichloromethane-d2 (99.9 atom% D, Euriso-top). Cyclohexane in technical grade were distilled before use. Triethylamine (≥99.5%, Roth) was dried over calcium hydride and subsequently distilled under argon. Tetrahydrofuran in HPLC grade (≥99.7%, VWR Chemicals) was dried over sodium and subsequently distilled under argon. Benzophenone was used to indicate the abstinence of water and oxygen.

1.2 Equipment

NMR: NMR spectra were recorded on a WB Bruker AVANCE I spectrometer operating at 500 MHz for 1H- and 125 MHz for 13C-measurement. CD2Cl2 was used as solvents and the resonance signal serves as reference for the chemical shift δ: 1H: CD2Cl2 = 5.32 ppm; 13C: CD2Cl2 = 54.00 ppm.

TLC: All thin layer chromatography experiments were performed on silica gel coated aluminium foil (silica gel 60 F254, Aldrich) or aluminium oxide coated aluminium foil (aluminium oxide 60 F254 neutral, Aldrich). The spots of reactants and product were visualized by irradiation with UV-lamp (256 nm and 365 nm) or by staining with Seebach-solution (mixture of phosphomolybdic acid hydrate, cerium(IV)-sulphate, sulfuric acid and water).

SEC: Size exclusion chromatography was performed on a Varian 390-LC gel permeation chromatography (GPC) system equipped with a LC-290 pump (Varian), refractive index detector (24 °C), PL AS RT GPC-autosampler (Polymer laboratories) and a Varian Pro Star column oven Model 510, operating at 40 °C. For separation, two systems were used. System A consisted of two SDV 5 µm linear S columns (8 x 300 mm) and a guard column (8 x 50 mm). System B consisted of two SDA 3 µm linear S columns (8 x 300 mm) and a guard column (8 x 50 mm).

IR: Infrared spectra were recorded on a Bruker Alpha-p instrument in a frequency range from 3,997.21 to 373.94 cm-1 applying ATR-technology (attenuated total reflection).

Orbitrap Electrospray-Ionization Mass Spectrometry (ESI-MS): mass spectra were recorded on a Q Excative (Orbitrap) mass spectrometer (Thermo Fisher Scientific, San Jose, CA, USA) equipped with an atmospheric pressure ionization source operating in the nebulizer assisted electrospray mode. The instrument was calibrated in the m/z-range 150-2,000 using premixed calibration solutions. A constant spray voltage of 3.5 kV and a dimensionless sheath gas of 6. The capillary voltage and the S-lens RF level were set to 68.0 V and 320 °C, respectively.

FAB: Fast atom bombardment mass spectra were recorded on a Finnigan MAT 95 instrument. The protonated molecular ion is expressed by the term: [(M+H)]+.

1.3 Synthesis

The synthesis of the oligomers (monomer 1, trimer 3, pentamer 5) attached to the TADF-oligomer conjugates was performed as described in Schneider et al. (2018).

1.3.1 Synthesis of (2r,3s,4s,6s)-2,3,4,6-tetra(9H-carbazol-9-yl)-5-(5-(4-((4-(phenylethynyl)-2,5-dipropoxyphenyl)ethynyl)phenyl)-1,3,4-oxadiazol-2-yl)benzonitrile T1

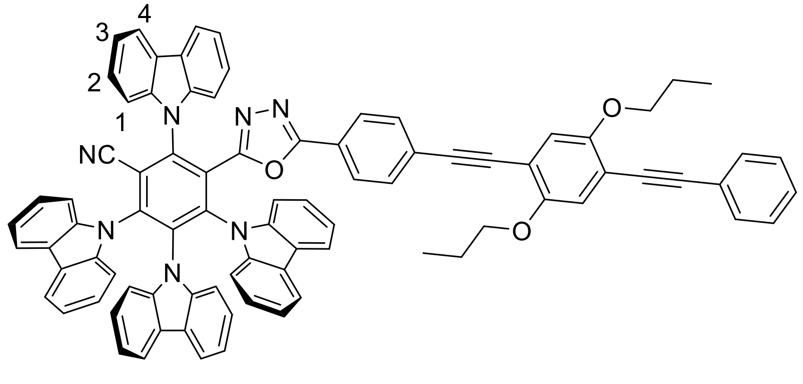

(2r,3s,4s,6s)-2,3,4,6-Tetra(9H-carbazol-9-yl)-5-(5-(4-iodophenyl)-1,3,4-oxadiazol-2-yl)benzonitrile **T** (50.0 mg, 48.4 µmol, 1.00 eq.), 1-ethynyl-4-(phenylethynyl)-2,5-dipropoxybenzene **monomer 1** (61.6 mg, 0.194 mmol, 4.00 eq.), 10 mol% *bis*(triphenylphosphine) palladium(II) dichloride (3.4 mg, 4.84 µmol) and 2.5 mol% copper(I) iodide (0.2 mg, 1.21 µmol) were placed into a Schlenk flask and evacuated three times. Under continuous argon flow, 20 mL dry THF and 100 µL dry triethylamine were added. The reaction mixture was stirred for 72 hours (3 days) at 45 °C, taken up in dichloromethane and washed with saturated NH_4Cl solution. The aqueous phase was extracted three times with dichloromethane. The combined organic layers were dried over Na_2SO_4, filtered and concentrated under reduced pressure. The residue was purified by silica column chromatography (dichloromethane/cyclohexane 2:3→8:1) to yield the product as a yellow solid (53.6 mg, 90%). TLC (dichloromethane/cyclohexane 3:1) R_f = 0.32; ^{1}H NMR (CD_2Cl_2, 500 MHz): δ (ppm) = 8.18 (d, J = 7.8 Hz, 2 H, carbazole 4), 7.89-7.76 (m, 2 H, carbazole 4), 7.70 (dd, J = 6.3, 2.2 Hz, 2 H, carbazole 4), 7.66-7.53 (m, 6 H, 2 C$\mathbf{H}_{\mathbf{aromatic}}$C-C≡C benzene end unit, carbazole 1, carbazole 2),

7.51-7.36 (m, 7 H, 3 C**H**aromatic benzene end unit, carbazole 3, carbazole 4), 7.36-7.28 (m, 4 H, 2 C**H**aromaticC-C≡C, carbazole 1), 7.26 (dd, *J* = 6.8, 1.7 Hz, 2 H, carbazole 1), 7.21-7.11 (m, 6 H, 2 C**H**aromaticC-C=N, carbazole 3, carbazole 1), 7.11-7.01 (m, 6 H, 2 C**H**aromaticCO, carbazole 2, carbazole 3), 6.90 (t, *J* = 7.4 Hz, 2 H, carbazole 3), 6.83-6.69 (m, *J* = 16.1, 8.1 Hz, 4 H, carbazole 2, carbazole 2), 4.02 (td, *J* = 6.4, 1.9 Hz, 4 H, 2 C**H**$_2$O), 1.99-1.78 (m, 4 H, 2 C**H**$_2$CH$_3$), 1.13 (t, *J* = 7.4 Hz, 6 H, 2 C**H**$_3$); ^{13}C NMR (CD_2Cl_2, 125 MHz): δ (ppm) = 164.62, 157.32, 154.35, 154.10, 144.18, 143.37, 142.53, 141.70, 139.78, 139.01, 138.30, 137.73, 132.02, 129.01, 128.57, 127.59, 127.42, 126.91, 126.45, 126.24, 125.44, 124.70, 124.51, 124.25, 123.79, 122.20, 122.13, 121.86, 121.76, 121.55, 121.41, 120.84, 120.80, 120.18, 118.74, 117.37, 117.31, 115.19, 113.51, 112.71, 110.76, 110.56, 110.02, 109.96, 95.58, 94.09, 89.82, 86.33, 71.61, 71.52, 23.26, 23.23, 10.90, 10.89; FAB of $C_{85}H_{57}N_7O_3$ (M+H$^+$ = 1224.5); IR (ATR) ν = 2960.6, 1599.9, 1479.5, 1443.9, 1333.0, 1309.0, 1216.3, 1150.7, 1013.8, 843.5, 741.8, 720.9, 688.9, 617.4, 527.6, 421.1, 385.0 cm^{-1}.

1.3.2 Synthesis of (2r,3s,4s,6s)-2,3,4,6-tetra(9H-carbazol-9-yl)-5-(5-(4-((4-((4-((4-(phenylethynyl)-2,5-dipropoxyphenyl)ethynyl)-2,5-dipropoxyphenyl)ethynyl)-2,5-dipropoxyphenyl)ethynyl)phenyl)-1,3,4-oxadiazol-2-yl)benzonitrile T3

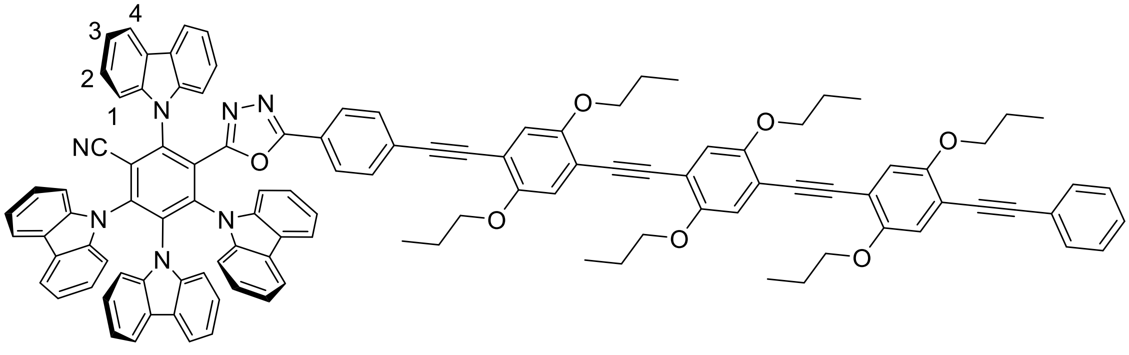

(2r,3s,4s,6s)-2,3,4,6-Tetra(9H-carbazol-9-yl)-5-(5-(4-iodophenyl)-1,3,4-oxadiazol-2-yl)benzonitrile **T** (50.0 mg, 48.4 µmol, 1.00 eq.), 1-ethynyl-4-((4-((4-(phenylethynyl)-2,5-dipropoxyphenyl)ethynyl)-2,5-dipropoxyphenyl)ethynyl)-2,5-dipropoxybenzene **trimer 3** (72.6 mg, 96.8 µmol, 2.00 eq.), 10 mol% *bis*(triphenylphosphine)palladium(II) dichloride (3.4 mg, 4.84 µmol) and 2.5 mol% copper(I) iodide (0.2 mg, 1.21 µmol) were placed into a Schlenk flask and evacuated three times. Under continuous argon flow, 20 mL dry THF and 100 µL dry triethylamine were added. The reaction mixture was stirred for 66 hours at 45 °C, taken up in dichloromethane and washed with saturated NH_4Cl solution. The aqueous phase was extracted three times with dichloromethane. The combined organic layers were dried over Na_2SO_4, filtered and concentrated under reduced pressure. The residue was purified by silica column chromatography (dichloromethane/cyclohexane 3:1→8:1) to yield the product as a yellow solid (70.3 mg, 88%). TLC (dichloromethane/cyclohexane 4:1) R_f = 0.24; ^{1}H NMR (CD_2Cl_2, 500 MHz): δ (ppm) = 8.16 (d, *J* = 7.8 Hz, 2 H, carbazole 4), 7.84-7.75 (m, 2 H, carbazole 4), 7.69 (dt, *J* = 6.4, 2.7 Hz, 2 H, carbazole 4), 7.63-7.57 (m, 2 H, carbazole 2), 7.57-7.51 (m, 4 H, 2 C**H**aromaticC-C≡C benzene end unit, carbazole 1), 7.49-7.36 (m, 7H, 3 C**H**aromatic benzene end unit, carbazole 3, carbazole 4), 7.33-7.25 (m, 4 H, 2 C**H**aromaticC-C≡C, carbazole 1), 7.25-7.19 (m, 2 H, carbazole 1), 7.17-7.09 (m, 6 H, 2 C**H**aromaticC-C=N, carbazole 3, carbazole 1), 7.09-6.99 (m, 10 H, 6 C**H**aromaticCO, carbazole 2, carbazole 3), 6.88 (t, *J* = 7.5 Hz, 2 H, carbazole 3), 6.74 (dd, *J* = 16.5, 7.9 Hz, 4 H, carbazole 2, carbazole 2), 4.09-3.95 (m, 12 H, 6 C**H**$_2$O), 1.96-1.79 (m, 12 H, 6 C**H**$_2$CH$_3$), 1.17-1.04 (m, 18 H, 6 C**H**$_3$); ^{13}C NMR (CD_2Cl_2, 125 MHz): δ (ppm) = 164.61,

157.31, 154.33, 154.13, 153.97, 153.93, 153.91, 144.17, 143.36, 142.52, 141.70, 139.78, 139.00, 138.30, 137.73, 132.00, 128.99, 128.94, 128.57, 127.58, 127.41, 126.90, 126.45, 126.24, 125.44, 125.38, 124.70, 124.50, 124.25, 123.88, 122.19, 122.12, 121.86, 121.76, 121.55, 121.39, 120.83, 120.78, 120.16, 118.74, 117.67, 117.64, 117.58, 117.49, 117.46, 115.37, 114.80, 114.54, 114.52, 114.49, 113.57, 112.70, 110.76, 110.56, 110.02, 109.96, 95.28, 94.17, 92.41, 92.12, 91.95, 91.88, 89.83, 86.47, 71.68, 71.65, 71.64, 71.61, 71.59, 71.51, 23.28, 23.24, 23.22, 10.89, 10.87; FAB of $C_{113}H_{89}N_7O_7$ ($M+H^+$ = 1658.0); IR (ATR) ν = 3049.6, 2961.7, 2872.9, 1599.1, 1489.5, 1444.7, 1421.2, 1381.8, 1333.2, 1309.2, 1273.3, 1209.2, 1150.7, 1119.1, 1060.8, 1012.3, 980.6, 844.1, 742.5, 721.1, 688.5, 617.5, 551.8, 527.9, 421.4 cm^{-1}.

1.3.3 Synthesis of (2r,3s,4s,6s)-2,3,4,6-tetra(9H-carbazol-9-yl)-5-(5-(4-((4-((4-((4-((4-((4-(phenylethynyl)-2,5-dipropoxyphenyl)ethynyl)-2,5-dipropoxyphenyl)ethynyl)-2,5-dipropoxyphenyl)ethynyl)-2,5-dipropoxyphenyl)ethynyl)-2,5-dipropoxyphenyl)ethynyl)phenyl)-1,3,4-oxadiazol-2-yl)benzonitrile T5

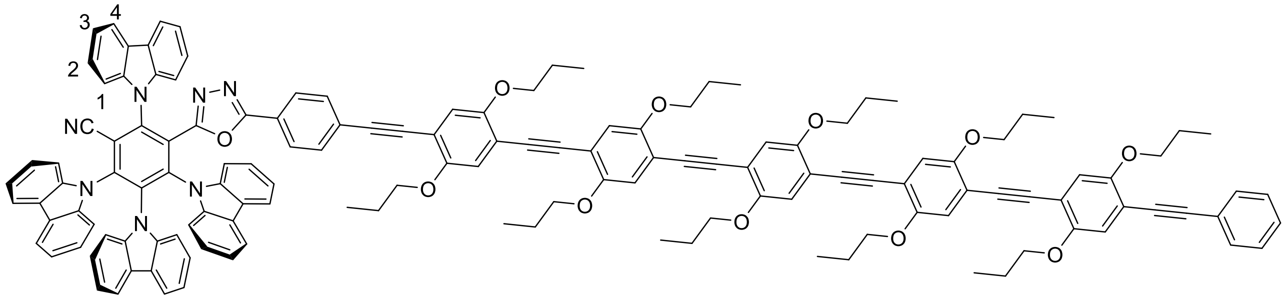

(2r,3s,4s,6s)-2,3,4,6-Tetra(9H-carbazol-9-yl)-5-(5-(4-iodophenyl)-1,3,4-oxadiazol-2-yl)benzonitrile **T** (50.0 mg, 48.4 µmol, 1.00 eq.), 1-ethynyl-4-((4-((4-((4-((4-(phenylethynyl)-2,5-dipropoxyphenyl)ethynyl)-2,5-dipropoxyphenyl)ethynyl)-2,5-dipropoxyphenyl)ethynyl)-2,5-dipropoxyphenyl)ethynyl)-2,5-dipropoxybenzene **pentamer 5** (115 mg, 96.8 µmol, 2.00 eq.), 10 mol% *bis*(triphenylphosphine)palladium(II) dichloride (3.4 mg, 4.84 µmol) and 2.5 mol% copper(I) iodide (0.2 mg, 1.21 µmol) were placed into a Schlenk flask and evacuated three times. Under continuous argon flow, 20 mL dry THF and 100 µL dry triethylamine were added. The reaction mixture was stirred for 74 hours at 45 °C, taken up in dichloromethane and washed with saturated NH_4Cl solution. The aqueous phase was extracted three times with dichloromethane. The combined organic layers were dried over Na_2SO_4, filtered and concentrated under reduced pressure. The residue was purified by silica column chromatography (dichloromethane/cyclohexane 4:1→1:0) and aluminium oxide column chromatography (pure dichloromethane) to yield the product as a yellow solid (35.5 mg, 35%). TLC (dichloromethane/cyclohexane 4:1) R_f = 0.22; ^1H NMR (CD_2Cl_2, 500 MHz): δ (ppm) = 8.16 (d, J = 7.8 Hz, 2 H, carbazole 4), 7.84-7.75 (m, 2 H, carbazole 4), 7.69 (dt, J = 6.4, 2.7 Hz, 2 H, carbazole 4), 7.63-7.57 (m, 2 H, carbazole 2), 7.57-7.51 (m, 4 H, 2 C**H**$_{\mathbf{aromatic}}$C-C≡C benzene end unit, carbazole 1), 7.49-7.36 (m, 7H, 3 C**H**$_{\mathbf{aromatic}}$ benzene end unit, carbazole 3, carbazole 4), 7.33-7.25 (m, 4 H, 2 C**H**$_{\mathbf{aromatic}}$C-C≡C, carbazole 1), 7.25-7.19 (m, 2 H, carbazole 1), 7.17-7.09 (m, 6 H, 2 C**H**$_{\mathbf{aromatic}}$C-C=N, carbazole 3, carbazole 1), 7.09-6.99 (m, 14 H, 10 C**H**$_{\mathbf{aromatic}}$CO, carbazole 2, carbazole 3), 6.88 (t, J = 7.5 Hz, 2 H, carbazole 3), 6.74 (dd, J = 16.5, 7.9 Hz, 4 H, carbazole 2, carbazole 2), 4.09-3.95 (m, 20 H, 10 C**H**$_2$O), 1.96-1.79 (m, 20 H, 10 C**H**$_2$CH$_3$), 1.17-1.04 (m, 30 H, 10 C**H**$_3$); ^{13}C NMR (CD_2Cl_2, 125 MHz): δ (ppm) = 164.62, 157.32, 154.34, 154.14, 153.96, 153.92, 144.17, 143.37, 142.53, 141.71, 139.78, 139.01, 138.31, 137.74,

132.00, 129.00, 128.94, 128.58, 127.59, 127.42, 126.91, 126.45, 126.25, 125.45, 124.71, 124.51, 124.25, 123.90, 122.20, 122.13, 121.86, 121.77, 121.55, 121.40, 120.84, 120.79, 120.17, 118.75, 117.66, 117.58, 117.50, 117.46, 115.37, 114.80, 114.70, 114.68, 114.64, 114.58, 114.51, 113.57, 112.71, 110.76, 110.56, 110.03, 109.97, 95.27, 94.18, 92.43, 92.18, 92.13, 92.08, 92.00, 91.91, 89.85, 86.50, 71.67, 71.65, 71.63, 71.58, 71.50, 23.28, 23.22, 10.88; ESI-MS of $C_{141}H_{121}N_7O_{11}$ ($[M+Na]^{2+}/2$ = 1067.45); IR (ATR) ν = 2919.9, 2850.0, 1721.9, 1599.1, 1490.5, 1453.2, 1422.0, 1384.0, 1334.0, 1310.8, 1273.2, 1209.2, 1061.2, 1013.2, 982.4, 859.3, 801.4, 743.9, 722.1, 617.6, 527.6, 426.2, 389.8 cm^{-1}.

2 Supplementary Figures

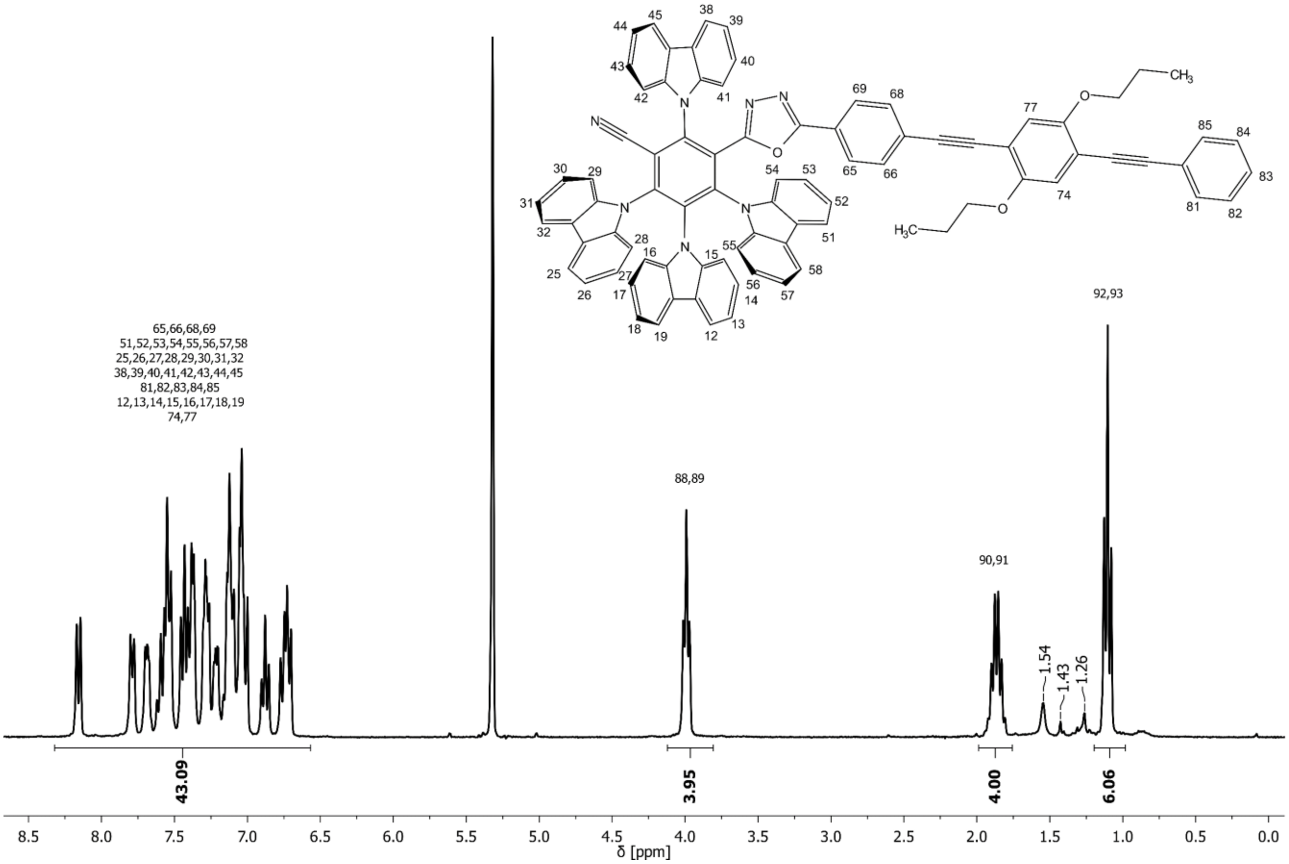

Supplementary Figure 1: ^{1}H NMR spectrum of monomer-TADF adduct **T1** with assigned signals.

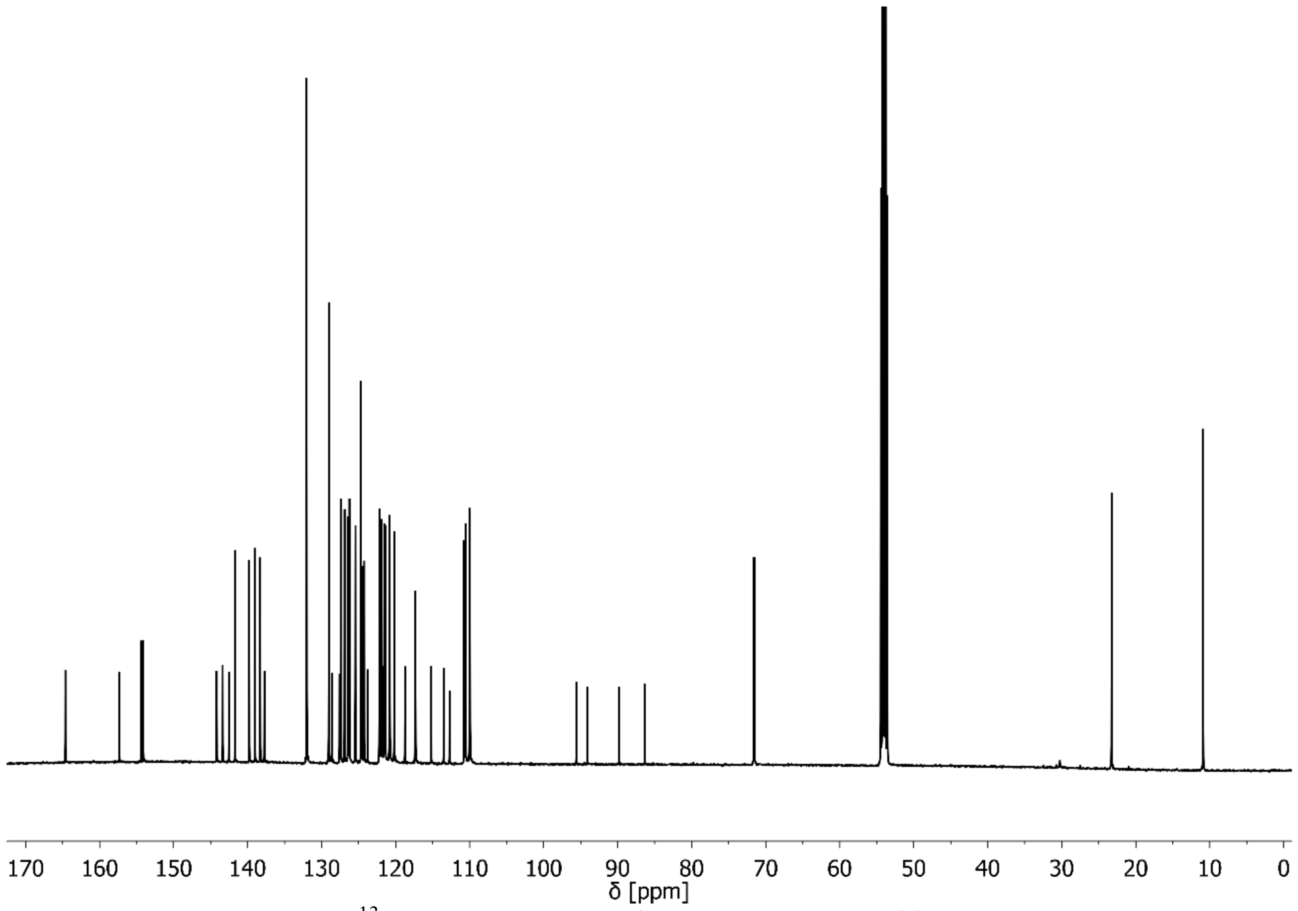

Supplementary Figure 2: ^{13}C NMR spectrum of monomer-TADF adduct **T1**.

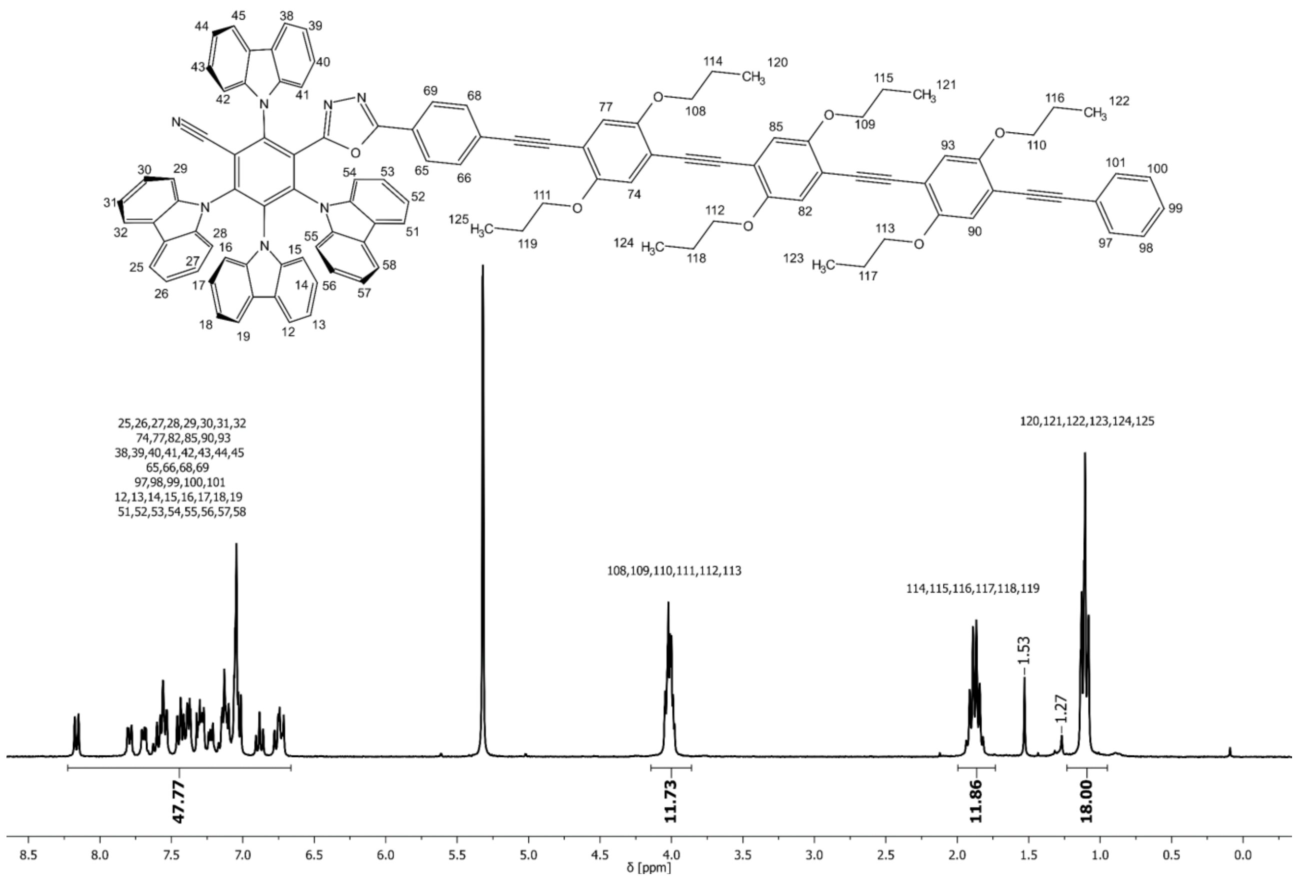

Supplementary Figure 3: ^{1}H NMR spectrum of trimer-TADF adduct **T3** with assigned signals.

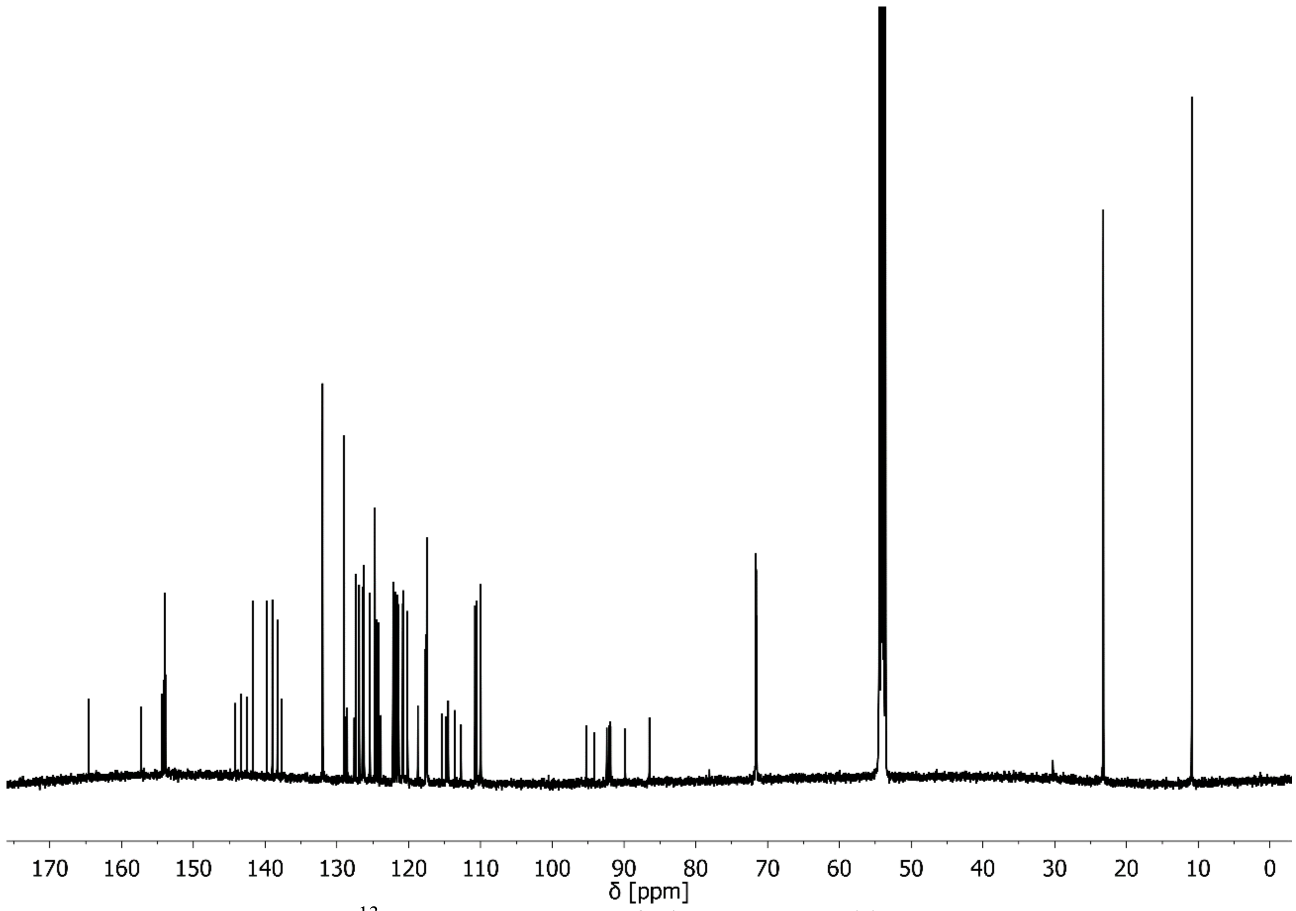

Supplementary Figure 4: ^{13}C NMR spectrum of trimer-TADF adduct **T3**.

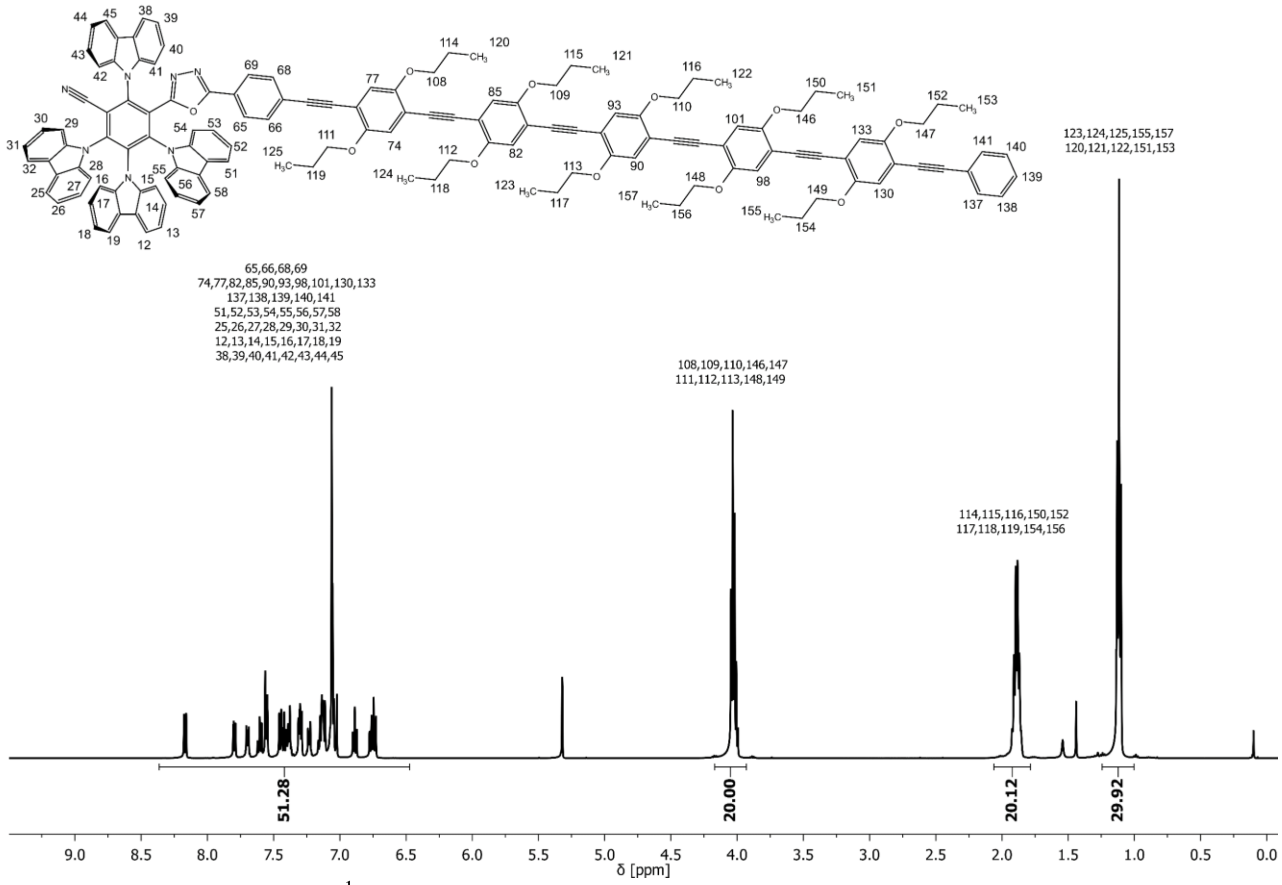

Supplementary Figure 5: ^{1}H NMR spectrum of pentamer-TADF adduct **T5** with assigned signals.

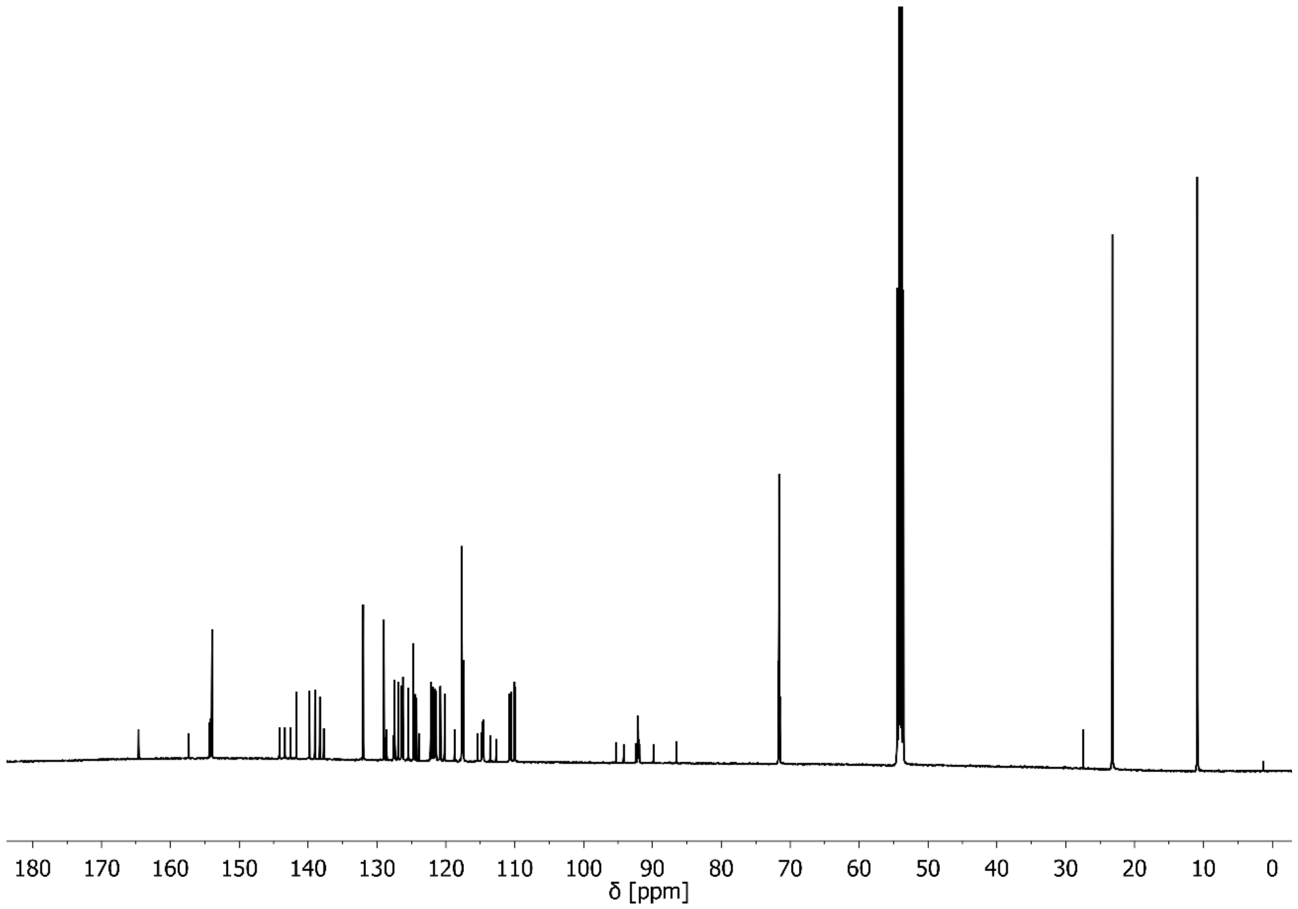

Supplementary Figure 6: ^{13}C NMR spectrum of pentamer-TADF adduct **T5**.

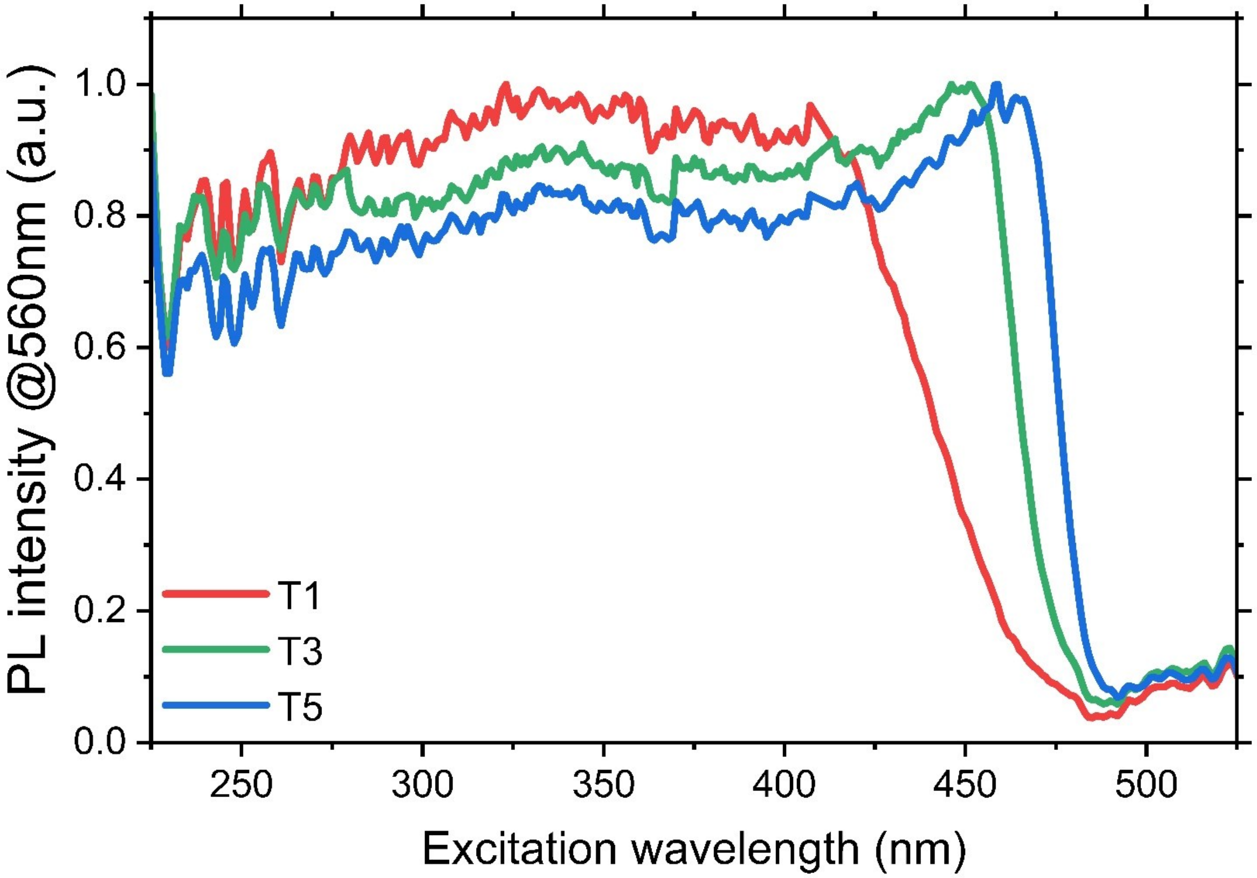

Supplementary Figure 7: Excitation spectra of the investigated compounds in deoxygenated chloroform solution ($c=5\cdot10^{-4}$ mol/l) monitoring the emission of the TADF core at 560 nm. The spectra are normalized to their maximum values (that correspond to absorption of the antenna). The emission of the TADF core after excitation of the antenna is clearly indicated by the peak that red-shifts with the oligomer length and matches the absorption of the oligomer.

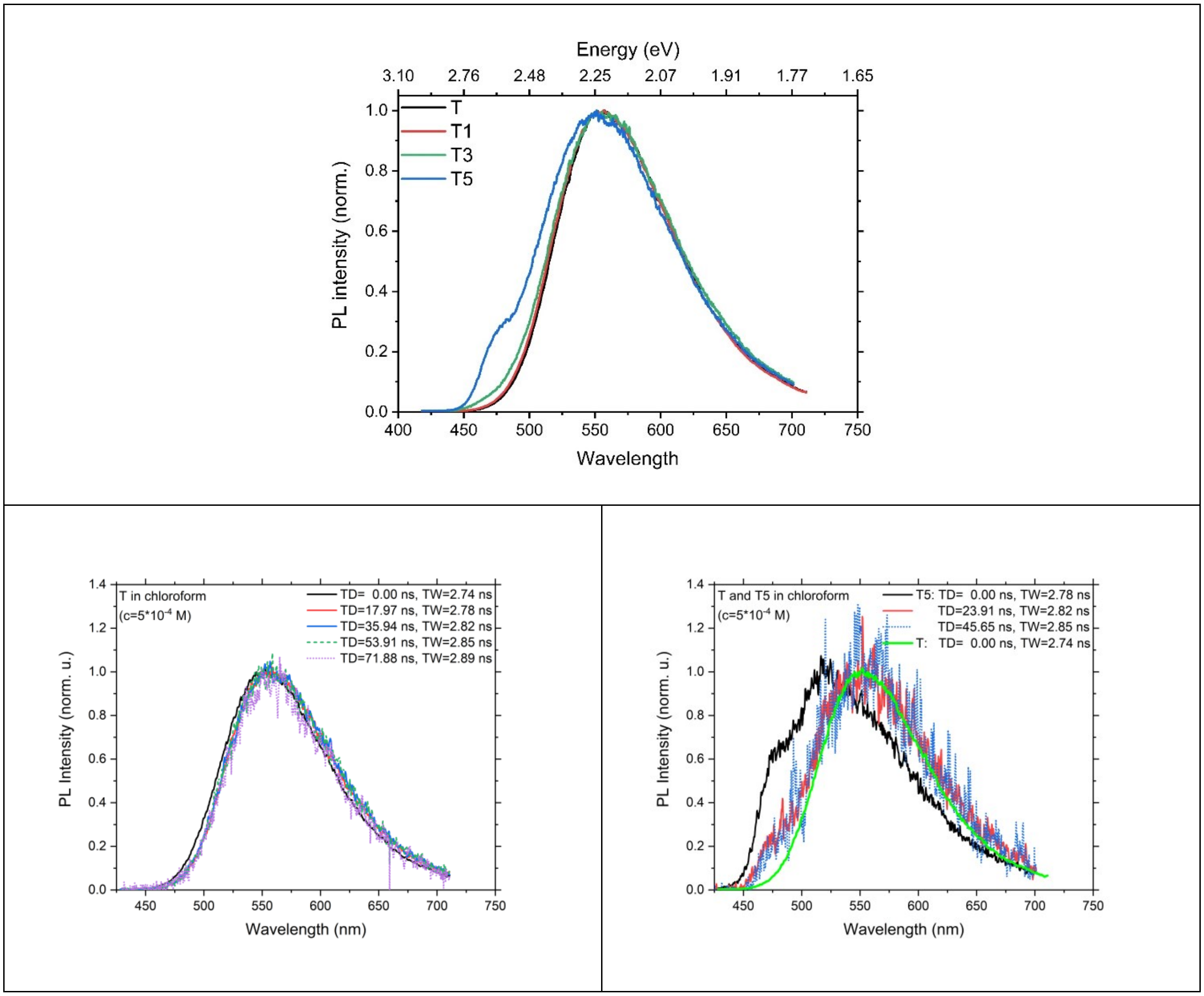

Supplementary Figure 8: Top Panel: Integrated prompt-only PL (accumulated up to 100 ns) of the investigated compounds in deoxygenated chloroform solution (c=$5\cdot10^{-4}$ mol/l). Exemplary spectral evolution of the prompt fluorescence for the extremes of the sequence, T (bottom left panel) and T5 (bottom right panel). The emission of the TADF core remains centered around 560 nm at late times, excluding the possibility of a lower-energy aggregate state. The shoulder around 475 nm present at short times for T5 is due to some emission from the oligomer antenna in this sample, as discussed in the main manuscript. Once the emission is dominated by the TADF core, no further spectral shift is observed, again indicating that the molecules are not aggregated. The spectral shape of the PL does not change as a function of time for T, excluding the possibility of an aggregated state being present in T. For T5, the spectra does initially shift as at early times some emission comes from the antenna. At late times for T5, the emission only comes from the TADF core, and its spectra well-matches that of T. This shows that aggregation does not play a role for the TADF-antenna conjugates (along with the similarity of the emission spectra for all other conjugates).

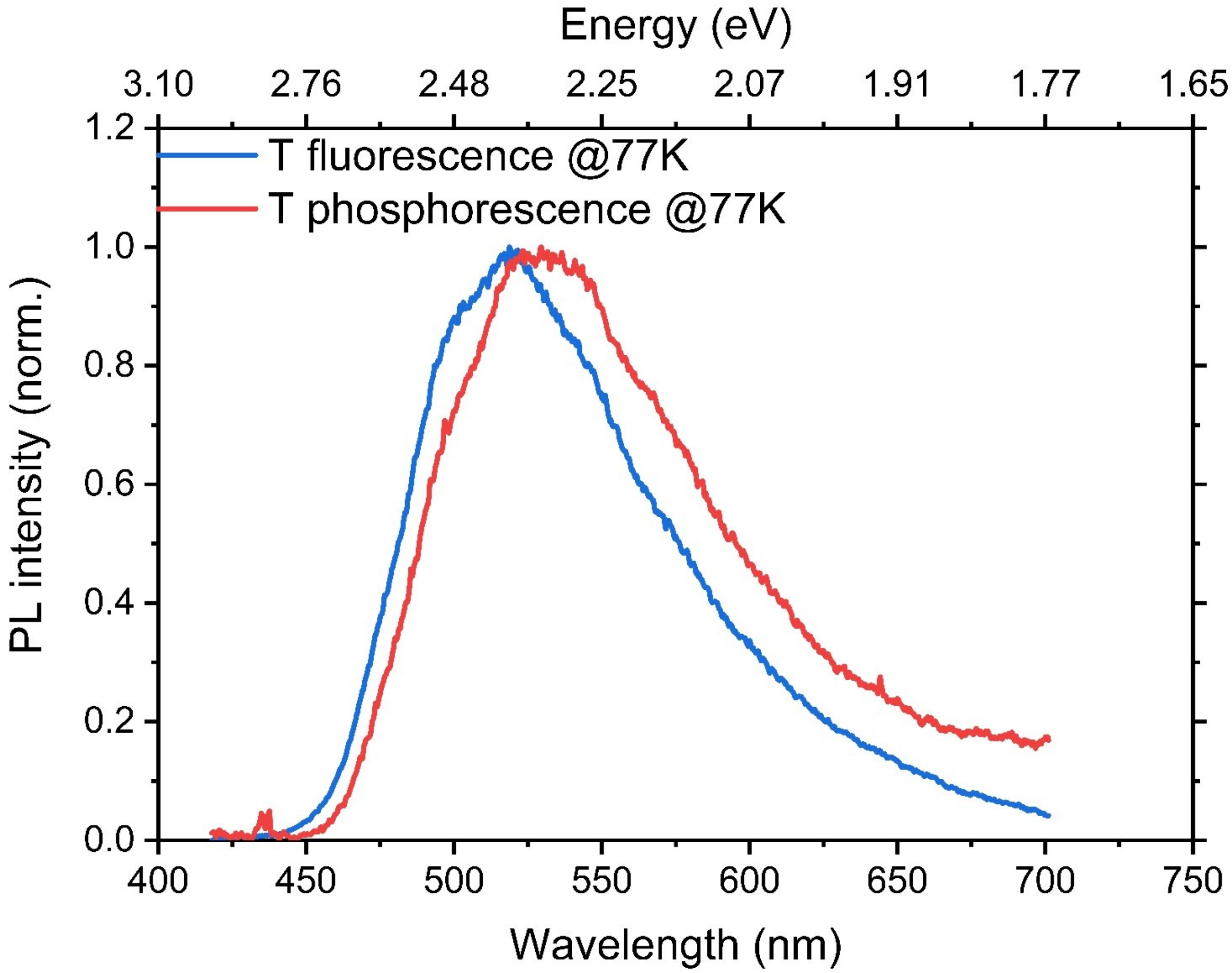

Supplementary Figure 9: Integrated prompt-only PL (fluorescence, accumulated up to 100 ns) and delayed-only PL (phosphorescence, accumulated from 500 ns to 190 µs) of **T** in deoxygenated chloroform solution (c=5·10-4 mol/l) at 77 K. From the onsets of the fluorescence and phosphorescence spectra, we calculated the value of the singlet-triplet splitting, ΔE_{ST}=0.02 eV.

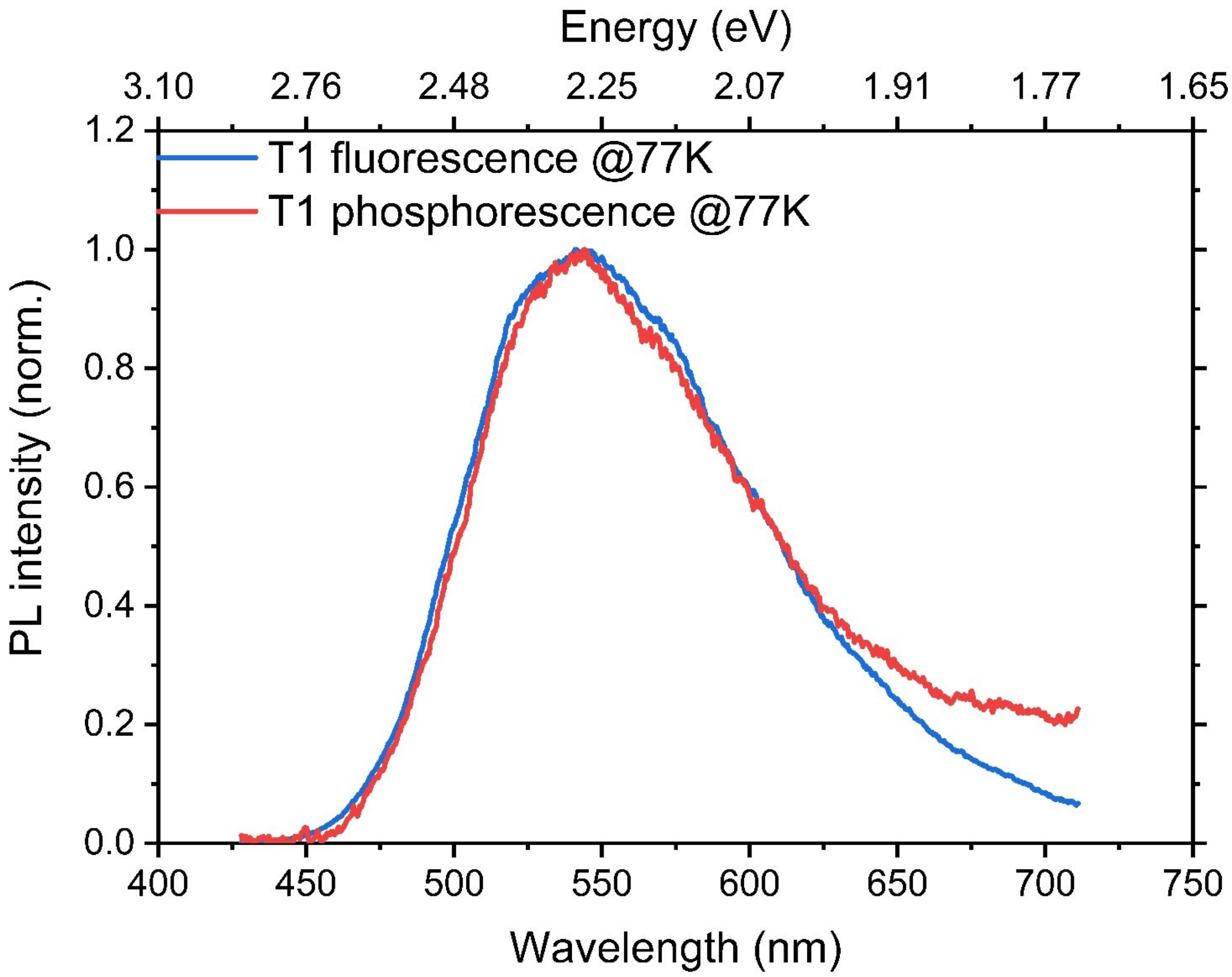

Supplementary Figure 10: Integrated prompt-only PL (fluorescence, accumulated up to 100 ns) and delayed-only PL (phosphorescence, accumulated from 500 ns to 190 µs) of **T1** in deoxygenated chloroform solution (c=5·10-4 mol/l) at 77 K. From the onsets of the fluorescence and phosphorescence spectra, we calculated the value of the singlet-triplet splitting, ΔE_{ST}=0.01 eV.

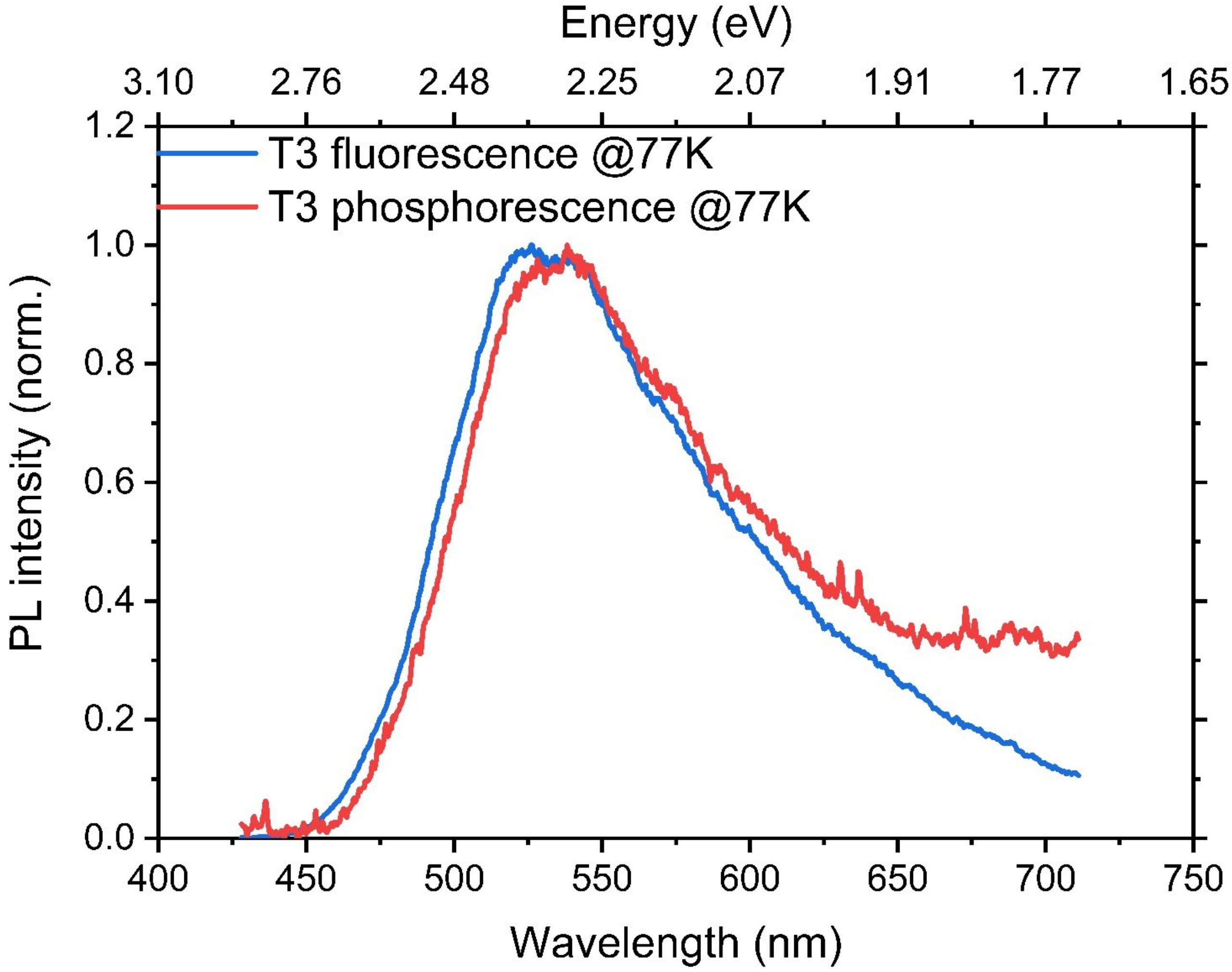

Supplementary Figure 11: Integrated prompt-only PL (fluorescence, accumulated up to 100 ns) and delayed-only PL (phosphorescence, accumulated from 500 ns to 190 μs) of **T3** in deoxygenated chloroform solution (c=5·10-4 mol/l) at 77 K. From the onsets of the fluorescence and phosphorescence spectra, we calculated the value of the singlet-triplet splitting, ΔE_{ST}=0.02 eV.

References

Schneider, R. V., Waibel, K. A., Arndt, A. P., Lang, M., Seim, R., Busko, D., et al. (2018). Sequence-definition in stiff conjugated oligomers. Scientific reports 8, 17483. doi:10.1038/s41598-018-35933-z
